# Traditional Chinese medicine for functional gastrointestinal disorders and inflammatory bowel disease: narrative review of the evidence and potential mechanisms involving the brain-gut axis

**DOI:** 10.3389/fphar.2024.1444922

**Published:** 2024-09-17

**Authors:** RuiXuan Liu, YunTian Luo, JinYing Ma, Qi Zhang, Yudong Sheng, Jiashan Li, Hongjiao Li, TianYi Zhao

**Affiliations:** ^1^ School of Traditional Chinese Medicine, Tianjin University of Traditional Chinese Medicine, Tianjin, China; ^2^ Institute of Basic Research in Clinical Medicine, China Academy of Chinese Medical Sciences, Beijing, China

**Keywords:** Traditional Chinese medicine, functional gastrointestinal disorders, inflammatory bowel disease, brain-gut axis, pharmacological mechanism, acupuncture

## Abstract

Functional gastrointestinal disorders (FGIDs) and inflammatory bowel disease (IBD) are common clinical disorders characterized by recurrent diarrhea and abdominal pain. Although their pathogenesis has not been fully clarified, disruptions in intestinal motility and immune function are widely accepted as contributing factors to both conditions, and the brain–gut axis plays a key role in these processes. Traditional Chinese Medicine (TCM) employs a holistic approach to treatment, considers spleen and stomach impairments and liver abnormality the main pathogenesis of these two diseases, and offers a unique therapeutic strategy that targets these interconnected pathways. Clinical evidence shows the great potential of TCM in treating FGIDs and IBD. This study presents a systematic description of the pathological mechanisms of FGIDs and IBD in the context of the brain–gut axis, discusses clinical and preclinical studies on TCM and acupuncture for the treatment of these diseases, and summarizes TCM targets and pathways for the treatment of FGIDs and IBD, integrating ancient wisdom with contemporary biomedical insights. The alleviating effects of TCM on FGID and IBD symptoms are mainly mediated through the modulation of intestinal immunity and inflammation, sensory transmission, neuroendocrine–immune network, and microbiota and their metabolism through brain–gut axis mechanisms. TCM may be a promising treatment option in controlling FGIDs and IBD; however, further high-quality research is required. This review provides a reference for an in-depth exploration of the interventional effects and mechanisms of TCM in FGIDs and IBD, underscoring TCM’s potential to recalibrate the dysregulated brain–gut axis in FGIDs and IBD.

## 1 Introduction

Functional gastrointestinal disorders (FGIDs) and inflammatory bowel disease (IBD) are characterized by recurrent abdominal discomfort, which can be accompanied by altered bowel regularity and stool properties (Black et al., 2020; Seyedian et al., 2019). In recent years, the increasing incidence of both diseases has garnered increasing attention from researchers. A recent study stated that in some countries in Latin America and Asia, such as Brazil, China, and India, IBD’s incidence will increase exponentially until reaching a stage of compounded prevalence in which morbidity exceeds mortality (Kaplan and Windsor, 2021). In addition, a cross-national prevalence study by the Rome Foundation revealed that >40% of the world’s population have FGIDs (Sperber et al., 2021). This places a heavy economic burden and a significant health risk on humanity. The Rome IV Criteria classify FGIDs into eight categories, and this paper focuses on the two most common FGIDs, namely, irritable bowel syndrome (IBS) and functional dyspepsia (FD) (Sperber et al., 2021), and IBDs, namely, ulcerative colitis (UC) and Crohn’s disease (CD). For FGIDs, existing treatments cannot identify and address disease pathogenesis but can only alleviate the patient’s gastrointestinal and psychological comorbidities (Black et al., 2020). In IBD research, routinely used therapeutic agents such as corticosteroids, aminosalicylates, and immunosuppressants (Hanauer and Baert, 1994) are still unable to cure IBD. In addition, individualized treatments with better efficacy and fewer side effects are still needed (Le Berre et al., 2023; Roda et al., 2020).

In as early as the 1840s, studies have found that emotions affect the digestion rate, confirming that a pathway exists between the gut and the nervous system (Margolis et al., 2021). Recent studies have shown a bidirectional interaction between the gut microbiota and the nervous system through direct and indirect mechanisms, which is known as the brain–gut axis. It encompasses the gut-associated immune system, enteric neuroendocrine system, enteric nervous system (ENS), central nervous system (CNS), etc. (Mayer et al., 2022). Patients with both FGIDs and IBD exhibited disruption of gut bacterial microbiota homeostasis (Bajaj et al., 2024; Ford et al., 2020a; Liu et al., 2017), resulting clinical symptoms. As regards mood states, two analyses have shown that >20% of patients with both disorders have concomitant symptoms of anxiety and depression and that more women than men are affected (Barberio et al., 2021; Jones et al., 2017). This proves that this link between the gut and the brain deserves more attention.

Traditional Chinese Medicine (TCM) has the potential to treat FGIDs and IBD. For example, studies have reported the effectiveness of acupuncture and moxibustion in treating IBS (Ma et al., 2024), and peppermint has been included in the 2021 clinical treatment guidelines for IBS by the American College of Gastroenterology (Lacy et al., 2021). Curcumin is also effective in improving gastrointestinal symptoms in IBS owing to its antioxidant and anti-inflammatory activities (Ng et al., 2018b). Herbal medicine is a safe and effective treatment of FD (Heiran et al., 2022), and sufficient evidence presents the utilization of modified Zhi Zhu decoction and Xiao Pi Kuan Wei decoction as viable alternatives for the treatment of individuals who do not respond to prokinetic agents (Ho et al., 2021). As an adjunctive treatment for UC, rhubarb combined with mesalazine or lorazepam is safer and more effective than when combined with Western medicine (Li Y. et al., 2022). Preclinical studies of UC have also shown sufficient evidence of the therapeutic effects of licorice extracts (Lu et al., 2022). The use of herbal medicines as complementary therapies in CD treatment has also been determined to be beneficial and may reduce the incidence of adverse events (Wang et al., 2019). These findings indicate the need to enhance TCM and its utilization as an alternative or complementary therapy for FGIDs and IBD. However, its mechanism of action has not yet been clarified, which limits its development and clinical promotion.

Herein, we outline the possible molecular mechanisms by which the brain–gut axis plays a role in FGIDs and IBD and integrate current evidence on the effects of TCM on these diseases in the context of the brain–gut axis. We also summarize the mechanism of action of TCM based on the brain–gut axis, laying the foundation for further in-depth exploration of the role and mechanism of TCM in FGIDs and IBD.

## 2 Exploring the pathogenesis of FGIDs and IBD in the context of the brain–gut axis

Understanding the pathogenesis of FGIDs and the brain–gut axis in IBD can aid in the development of clinical interventions for these diseases. Herein, we summarize the pathogenesis of these diseases in terms of immunity, enterosensory transmission, neuroendocrine–immune network (NEI), and intestinal flora.

### 2.1 FGIDs

In clinical practice, FGIDs are prevalent, and patients typically report a single symptom or an arbitrary mix of symptoms. The clinical symptoms of IBS are mainly recurrent abdominal pain associated with abnormal fecal patterns or frequency, and the pathogenesis is mainly associated to genetics, stress, dysregulation of intestinal microbial homeostasis, visceral hypersensitivity, and intestinal motility disorders (Ford et al., 2020b). The main FD symptoms include epigastric pain or burning sensations, postprandial fullness or early satiety, intestinal motility and sensory disturbances, gut microenvironmental disturbances, and immune-related inflammation (Ford et al., 2020a). Despite variations in the etiology and clinical features of FGIDs, current literature presents that FGID symptoms primarily arise from disrupted intestinal motility. Therefore, the research direction of FGIDs has focused on intestinal sensory transduction. Furthermore, substantial convergence exists in the underlying mechanisms of different FGIDs.

**FIGURE 1:**
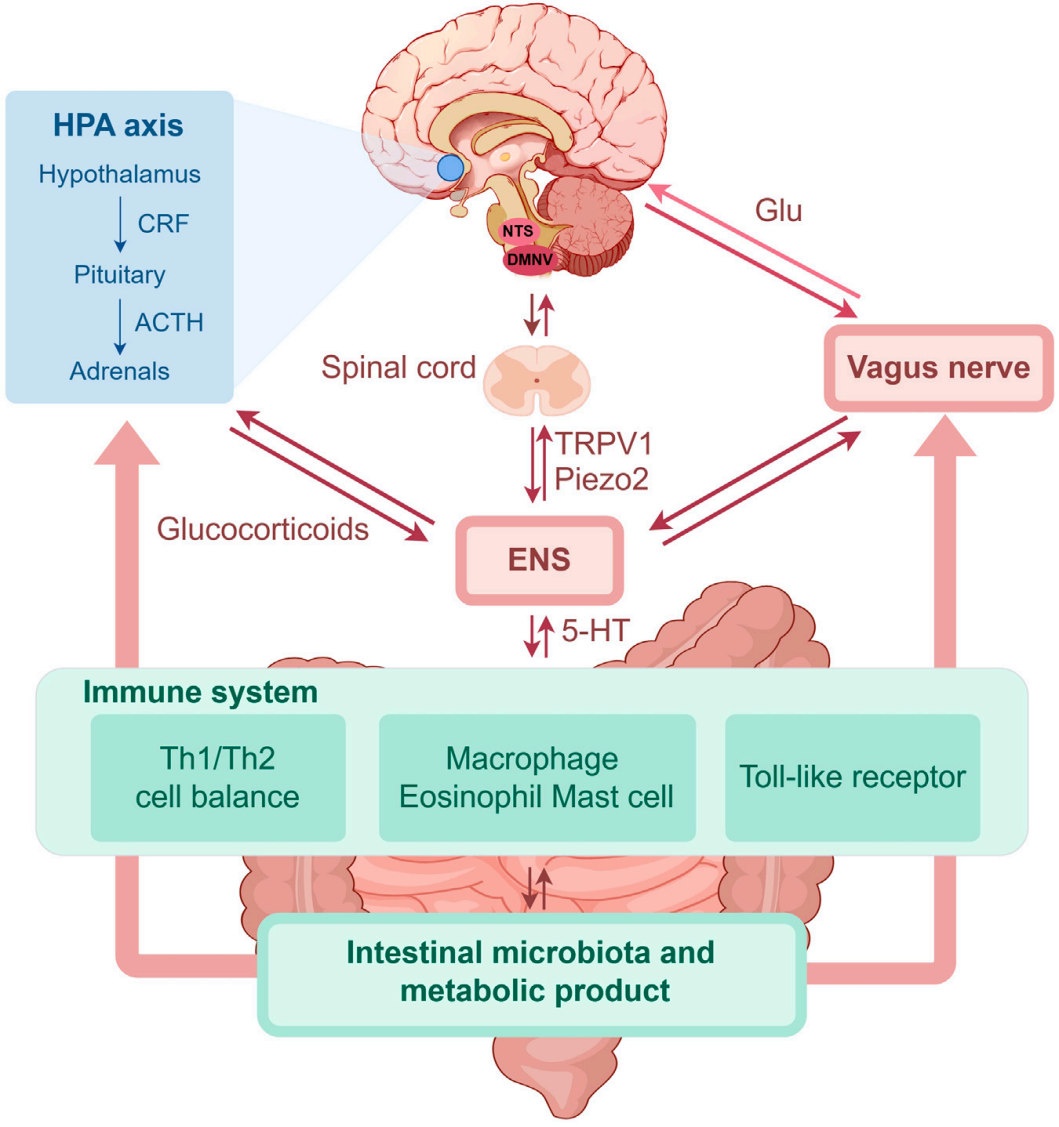
The role of brain-gut axis in FGIDs (by Figdraw). FGIDs, functional gastrointestinal disorders; HPA, hypothalamic-pituitary-adrenal; CRF, corticotropin releasing factor; ACTH, adrenocorticotropic hormone; NTS, nucleus of the solitary tract; DMNV, dorsal motor nucleus of the vagus; Glu, glutamate; ENS, enteric nervous system; 5-HT, 5-hydroxytryptamine; TRPV1, transient receptor potential vanilloid 1; Th, T helper.

Through the integration of previous studies on the brain–gut axis, we have compiled an overview of the potential pathogenesis of FGIDs (Figure 1).

#### 2.1.1 Intestinal mucosal immunity and inflammation

Researchers have proposed that low-grade inflammation, immune activation, and dysregulated gut flora homeostasis may contribute to IBS onset and exacerbation (Ng et al., 2018a; Rodríguez-Fandiño et al., 2010). In particular, gastrointestinal macrophages are actively involved in the intestinal immune response (Zhou S. Y. et al., 2023), are mainly responsible for phagocytosis and digestion, and promote tissue remodeling (Gentek et al., 2014).

T helper (Th) 1 cells activate macrophages to generate a cellular immune response, whereas Th2 cells inhibit this response (Butcher and Zhu, 2021). However, in FGIDs, a shift in immune cells to a Th2-type response may ensue to suppress low-grade inflammation (Du et al., 2019; Kindt et al., 2009). Furthermore, in the intestinal mucosa of patients with IBS, levels of Toll-like receptor (TLR) 4 and TLR5 expressed on eosinophils and mast cells are significantly higher than that in healthy individuals (Brint et al., 2011; Dlugosz et al., 2017), which exacerbates intestinal hypersensitivity and increases the release of mast cell degranulation (Mallaret et al., 2022; Yang et al., 2019). Overall, intestinal immune responses induced by abnormal macrophage activation, an imbalanced Th1 cell/Th2 cell ratio, and a high TLR expression, are closely associated with the pathogenesis of FGIDs.

#### 2.1.2 Intestinal sensory transduction

The human ENS consists of neurons and enteric glial cells, which independently innervate gastrointestinal activity and are connected to the CNS through the vagus nerve (VN) and the sympathetic and pelvic nerves (Furness et al., 2014). A study presented that the pathogenesis of FGIDs was inherently linked to visceral hypersensitivity, which was first elucidated five decades ago (Mertz et al., 1995; Ritchie, 1973).

Regarding the gut sensory afferent pathway, transient receptor potential vanilloid (TRPV) specifically expressed in the dorsal root ganglion in central sensitization takes on a crucial role in the transmission of nociceptive signals from the gut to the CNS (Farmer and Aziz, 2009). This ion channel is activated by stimuli, such as heat, acidity, and mechanical pressure, and is responsible for the detection and transmission of pain signals from the gut to the brain, making them a potential treatment target in gut-related pain disorders. A functional magnetic resonance imaging study of the brains of patients with IBS revealed the activation of the thalamus, insula, and anterior cingulate cortex, which confirms the presence of central sensitization in these patients (Price et al., 2007). TRPV1 and TRPV4 are closely associated with hypersensitivity in mouse experiments (Ho et al., 2012; Xie et al., 2023), and TRPV4 activation inhibits intestinal peristalsis by reducing NO-dependent Ca2^+^ release from enteric neurons (Fichna et al., 2015). The selective ablation of the Piezo (piezo-type echanosensitive ion channel component) 2 protein expressed in TRPV1-spectrum neurons attenuates visceral hypersensitivity in IBS mice (Xie et al., 2023), which explains why abdominal pain is one of the main IBS symptoms.

As regards visceral hypersensitivity, the role of the sympathetic nerves and the VN is still unclear. However, the stimulation of the auricular VN may be effective in relieving constipation and abdominal pain symptoms in patients with IBS (Shi et al., 2021). Similar studies have reported comparable results in a rat model of FD (Guo and Gharibani, 2024; Hou et al., 2023).

#### 2.1.3 NEI

The NEI theory emphasizes the coordinated interaction between the nervous, endocrine, and immune systems. It was first proposed in 1977. A study suggested that NEI dysregulation may be a significant contributing factor to the pathogenesis of IBS (Powell et al., 2017).

Reactivating neurons in the insular cortex of the brain restore their original inflammatory state (Koren et al., 2021), providing new ideas for suppressing peripheral inflammation. 5-hydroxytryptamine (5-HT), an important neurotransmitter in the CNS, is a potential research direction for IBS (Ford et al., 2024). Enterochromaffin cells (ECs) release 5-HT to activate the 5-HT_3_ receptor in intestinal submucosal neurons to transmit pain signals (Bellono et al., 2017; Diwakarla et al., 2017).

The VN is also a key link in the NEI of FGIDs, and the efferent fibers of the VN originated from the preganglionic neurons in the dorsal motor nucleus of the vagus and then descended to the postganglionic neurons in the ENS. The afferent fibers of the VN activate receptors on the nucleus of the solitary tract in the medulla oblongata primarily through the neurotransmitter glutamate, which transmits signals to higher brain regions (Jean, 2001). The dorsal motor nucleus of the vagus and the nucleus of the solitary tract are located adjacent to each other, and an experiment suggests that the two are connected, resulting in a vagal loop formation (Rinaman et al., 1989). Moreover, the afferent fibers of the VN can regulate inflammation by activating the hypothalamic–pituitary–adrenal (HPA) axis. Environmental stressors can also activate the HPA axis (Agirman et al., 2021; Pavlov et al., 2003). Corticotropin-releasing factor (CRF) 1 plays a pathogenic role in IBS, and CRF1 and CRF2 can regulate visceral hypersensitivity and intestinal permeability through the modulation of TLR4 and the cytokine system, a mechanism that can be blocked by interleukin (IL)-1 receptor antagonists. This mechanism is also positively regulated by lipopolysaccharide (Nozu et al., 2018). In the HPA axis, the adrenal cortex releases cortisol, which can directly activate immune cells such as lymphocytes in the gastrointestinal tract and promote peripheral sensitization. Cortisol binding to the amygdala further promotes the hypothalamic secretion of adrenocorticotropic hormones, exacerbating central sensitization (Moloney et al., 2016).

Extraintestinal macrophages also produce bone morphogenetic protein 2 that stimulates intestinal neurons to produce colony stimulating factor 1 to further increase the number of macrophages. This can be exacerbated by bacterial infections in the gastrointestinal tract. The positive feedback loop could play a role in IBS development (Kabata and Artis, 2019; Muller et al., 2014).

#### 2.1.4 The gut microbiota and metabolites

The gut microbiota helps maintain host health and intestinal homeostasis. IBS subtypes demonstrated varied bacterial composition (Carco et al., 2020). However, the uniform characterization of the IBS-associated gut microbiota remains elusive (Ng et al., 2023). An interaction exists between stress and gut flora (Li Q. et al., 2023; O'Mahony et al., 2017; Tannock and Savage, 1974). According to Mujagic et al. (2022), the level of psychological stress in patients and the magnitude of the effect of psychological stress on IBS symptoms can be used as a basis for clustering the fecal microbiome–metabolome characteristics of patients with IBS, demonstrating the existence of such interactions in IBS. Studies have indicated that the gut microbiota influences gut sensory transduction (Meynier et al., 2024; Shimbori et al., 2022; Vicentini et al., 2021), intestinal immunity (Gobert et al., 2016; Jia et al., 2019; Zheng et al., 2023), neuroendocrine pathways (Gao et al., 2022; Mishima and Ishihara, 2021), and intestinal motility (Gu et al., 2022; Shanahan et al., 2023). Furthermore, previous studies (Dodd et al., 2017; Wei et al., 2021; Yang W. et al., 2020) have demonstrated that the dysregulation of tryptophan metabolites, secondary bile acids, and short-chain fatty acids (SCFAs), are metabolites of the intestinal flora, affects normal intestinal immune function.

### 2.2 IBD

**FIGURE 2:**
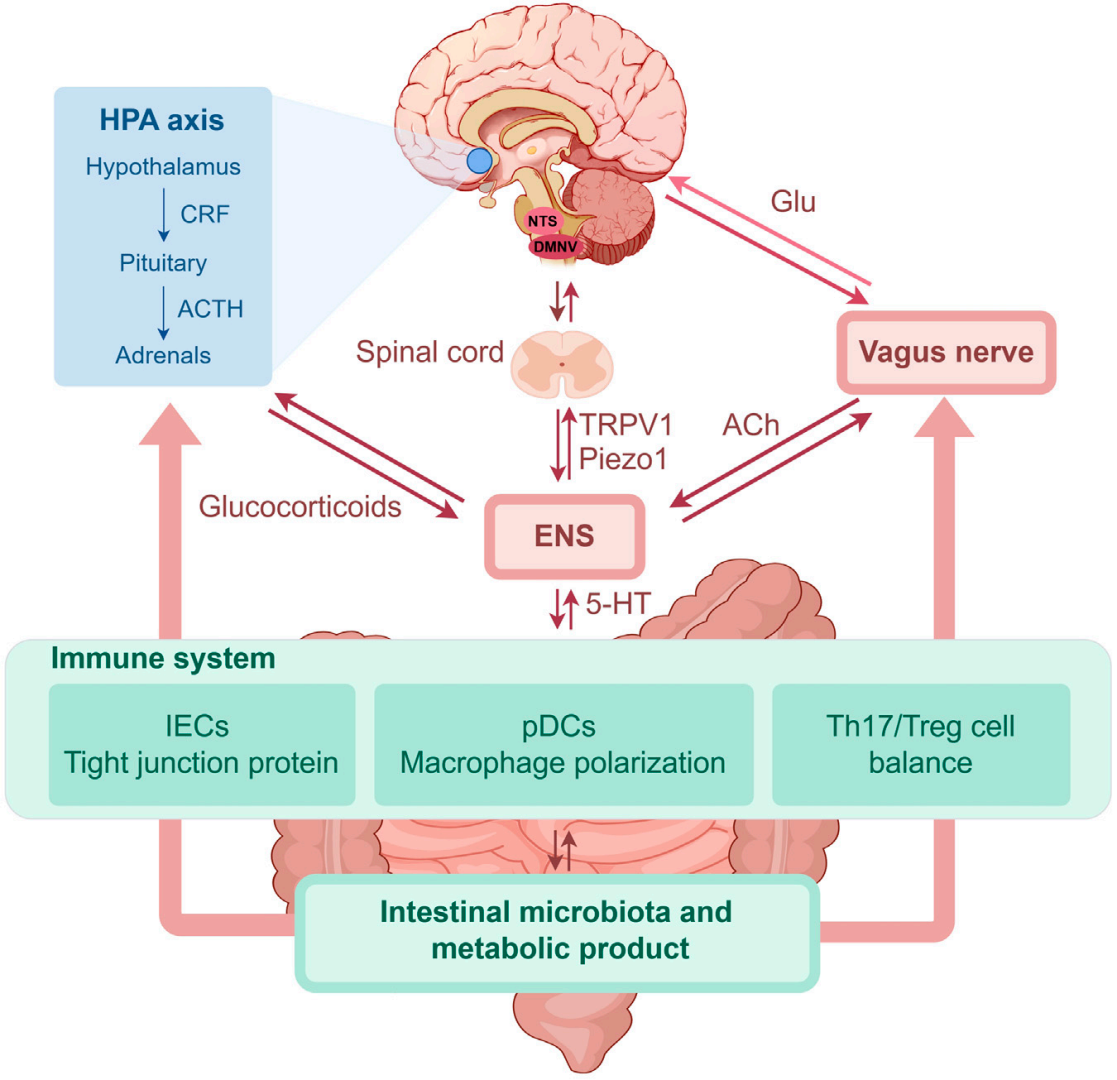
The role of brain-gut axis in IBD (by Figdraw). IBD, inflammatory bowel disease; HPA, hypothalamic-pituitary-adrenal; CRF, corticotropin releasing factor; ACTH, adrenocorticotropic hormone; NTS, nucleus of the solitary tract; DMNV, dorsal motor nucleus of the vagus; Glu, glutamate; ENS, enteric nervous system; 5-HT, 5-hydroxytryptamine; TRPV1, transient receptor potential vanilloid 1; ACh, acetylcholine; IECs, intestinal epithelium cells; pDCs, plasmacytoid dendritic cells; Th, T helper; Treg, regulatory T.

Symptoms of this chronic inflammatory disease of the gastrointestinal tract include abdominal pain, diarrhea, and rectal bleeding. Extraintestinal manifestations of IBD may also be observed in the skin, eyes, or joints; however, its etiology is unclear (Eberhardson et al., 2021; Rogler et al., 2021). Despite ongoing research, IBD has no known cure, and treatment options focus on symptom management and complication prevention. This section focuses on UC and CD. UC is a chronic nonspecific inflammatory disease that often leads to structural and functional changes in the colon and rectum, and its pathogenesis may be related to intestinal barrier disruption, intestinal immune abnormalities, and dysregulated intestinal homeostasis (Le Berre et al., 2023). CD, also a chronic inflammatory disease, affects the terminal ileum and proximal colon, and it may be caused by genetic factors, intestinal barrier disruption, and intestinal flora disorders (Dolinger et al., 2024). Although the inflammatory features and sites of CD differ from those of UC, immune response abnormalities and intestinal inflammation have been recognized as disease drivers. Most of the current studies on IBD also focus on this aspect. The summarized pathogenesis of IBD is shown in Figure 2.

#### 2.2.1 Mucosal immunity and inflammation in IBD

##### 2.2.1.1 Innate immunity in IBD

Intestinal epithelial cells (IECs) are the first line of defense in the intestine. These proteins are tightly packed together through cellular junctions, which nourish the gut and functions as an intestinal immune barrier (Berin et al., 2006; Deuring et al., 2013). Studies have revealed that the mitochondrial function of IECs is closely related to the intestinal flora, and their dysfunction contributes to the development of ileitis (Alula et al., 2023). The low expression of the protein zonula occludens-1 is a possible reason for the failure of mucosal healing in patients with IBD (Kuo et al., 2021).

Antigen-presenting cells encompass dendritic cells (DCs) and macrophages. Plasmacytoid DCs (pDCs) are one of two subtypes of human DCs that help in activating host innate and adaptive immunity (McKenna et al., 2005; Zhang et al., 2021). pDCs promote the mobilization of colonic phagocytes into inflamed intestinal tissues, leading to the development and exacerbation of acute colitis (Arimura et al., 2017). In addition to DCs, macrophages are also associated with IBD development and exacerbation. In mouse model experiments, enhancing the transformation of M1 macrophages into M2 alleviates experimental colitis (Zhu et al., 2016), whereas psychological stress promotes macrophage polarization toward the M1 phenotype and infiltration into the colon (Ge et al., 2022). The inhibition of M1 polarization mitigates intestinal mucosal inflammation (Chu et al., 2023; Ruan et al., 2023). Furthermore, alterations in sodium–potassium ATPase have been identified as an underlying factor in diarrhea among patients with IBD. In an *in vitro* study involving IECs, the activation of TLR2, TLR4, and TLR7 led to the downregulation of sodium–potassium ATPase, whereas TLR5 exhibited the opposite effect (Cosme et al., 2023).

##### 2.2.1.2 Adaptive immune in IBD

The imbalance in Th17/regulatory T (Treg) cells has emerged as a potential contributing factor to IBD development. Research indicates that the modulation of the extracellular signal-regulated kinase/mitogen-activated protein kinase (MAPK) pathway can potentially restore the homeostasis of Th17/Treg cells (Liu et al., 2013). Notably, the inhibition of the Harvey rat sarcoma viral oncogene homolog, an upstream pathway regulator, counteracted MAPK-induced enhancement of Th17 cell differentiation and reinstated Th17/Treg cell equilibrium (Wang S. et al., 2024).

#### 2.2.2 Intestinal sensory transduction

Psychological stress activates the sympathetic nerves and decreases the vagal tone, inhibits its anti-inflammatory effects, promotes TNF-α production by macrophages, and exacerbates colitis progression (Bonaz et al., 2016; Breit et al., 2018). Studies have indicated that patients who underwent vagotomy have a lower risk of IBD development (Liu B. et al., 2020) and that electrical stimulation of the VN can improve clinical symptoms in patients with IBD (Sahn et al., 2023). TRPV1^+^ afferent signaling induces visceral hypersensitivity in colitis mice by preventing microglial activation in the spinal cord (Defaye et al., 2022). The activation of Piezo channels is crucial in the pathogenesis of IBD, particularly in CD. In the ileal mucosal tissues of patients with CD, high levels of Piezo1 protein expression leads to the activation of NOD-like receptor family pyrin domain containing (NLRP) 3 inflammasomes, which cause intestinal inflammation (Liu Q. et al., 2023). Vascular inflammation is widely recognized as a prominent issue in individuals with CD, and its onset is believed to be associated with the protracted exposure of vascular endothelial cells to shear stress. The deleterious effects of shear stress on the endothelium closely intertwined with the activation of calcium signaling mediated by the Piezo1 protein, ultimately resulting in the opening of TRPV4 channels (Hartmannsgruber et al., 2007; Swain and Liddle, 2021).

#### 2.2.3 NEI

Similar to the neuroimmune mechanism of FGIDs, EC and 5-HT are highly expressed in dextran sulfate sodium (DSS)-induced rat colonic mucosa (Oshima et al., 1999). In recent studies, the expression of 5-HT_7_ receptor has increased in UC and CD, and the 5-HT_7_ receptor may play a key role in IBD progression (Wan et al., 2023; Xu Z. et al., 2022). In the HPA axis, excessive stress-induced release of glucocorticoids and adrenocorticotropic hormones disrupts tight junction proteins between IECs, which weakens the intestinal mucosal barrier (Xu W. et al., 2021; Zong et al., 2019). The cholinergic anti-inflammatory pathway has also been implicated in the pathogenesis of IBD (Andersson and Tracey, 2012; Borovikova et al., 2000). In a mouse model of DSS- and dinitrobenzene sulfonic acid-induced colitis, attenuating the alpha7 nicotinic acetylcholine receptor-mediated release of macrophage inflammatory factors from the VN was found to result in the reactivation of intestinal inflammation, demonstrating that the cholinergic anti-inflammatory pathway induces intestinal inflammation (Ghia et al., 2009; Yasuda et al., 2011). Intestinal inflammation also increases intestinal permeability, which allows harmful substances in the intestine to enter the CNS by disrupting the gut vascular barrier and blood–brain barrier, which in turn stimulates immune cells in the CNS to trigger a neuroinflammatory response and exacerbates the inflammatory response at the central and peripheral levels (Agirman et al., 2021).

#### 2.2.4 Gut microbial metabolites

Gut microbiota dysbiosis is also involved in the pathogenesis of IBD. The composition of the mucosal microbiota in patients with IBD can influence the expression of mucosal inflammatory genes and intestinal cell types and be used to demonstrate the existence of mucosal host–microbe interactions in IBD (Hu et al., 2024). The concept of IECs–microbiota–immune cell crosstalk has also been proposed (Iyer et al., 2023). The bile acid metabolites 3-oxolithocholic acid and isolithocholic acid inhibited Th17 cell differentiation, demonstrating that they modulate the Th17/Treg cell balance and influence intestinal immunity (Paik et al., 2022). Recent studies have demonstrated that the intestinal flora affects the level of conjugated linoleic acid in the mouse intestine, which upregulates IL-18 signaling in intraepithelial lymphocytes and promotes the differentiation of CD4^+^ CD8αα^+^ intraepithelial lymphocytes (Song et al., 2023).

## 3 Understanding the brain–gut axis, FGIDs, and IBD in TCM

The concept of internal organs in TCM is different from that in modern medicine, the heart of TCM includes a part of the function of the brain in modern medicine, and the small intestine includes a part of the intestines. In TCM, a theory revealed that the heart and the small intestine are related exteriorly interiorly, which also corresponds well to the brain–gut axis nowadays. A core of TCM is the overall concept, advocating the integration of human and universe, that is, every internal organ in the human body is interconnected. Functionally, the intestines produced by the refined essence of the material also need to replenish the essence and marrow of the brain. The essence of the brain can also nourish the internal zang-fu located in the lower part of the body, which also coincides with the brain–gut axis.

In TCM, FGIDs may be described as gastric distension, abdominal pain, and diarrhea. IBD may be an intestinal abscess and dysentery. The spleen (pi) and stomach (wei) are the hubs of Qi ascending and descending, and spleen and stomach malfunction is considered the main cause of the disease. In TCM, the stomach is the main organ involved in the preliminary processing and digestion of food, after further conduction to the intestines, the main descending adversely risen Qi, whereas the spleen further transforms food into the body’s refined essence and transported to the whole body, the main ascending Qi. The normal synergy of the two can help the human body Qi and blood abundant, and maintain good health (Zhang et al., 2024). In the case of spleen and stomach dysfunction, the essence cannot nourish the limbs and zang-fu, and disturbed Qi activity, bloating, diarrhea, and pain will occur. Over time, the disturbed Qi activity will also lead to the production of pathological products such as dampness and blood stasis, aggravating the impeding of Qi activity. The liver also plays a role in pathogenesis, as it regulates the smooth flow of Qi, and liver disease can result in similar pathologic changes as described above. Therefore, the treatment of FGIDs by TCM mainly aims at restoring the normal physiology of the liver, spleen, and stomach and adopts different treatments according to symptoms, such as harmonizing the liver and spleen, invigorating the spleen, and harmonizing the stomach. Meanwhile, IBD treatment is directed at moving Qi to remove stagnancy and obstruction, clearing heat and resolving dampness, and promoting blood circulation to remove blood stasis.

## 4 Clinical efficacy and safety of TCM in FGIDs and IBD

TCM has accumulated rich clinical experience in the treatment of FGIDs and IBD and has demonstrated relatively clear efficacy in improving symptoms and quality of life of patients (Chiarioni et al., 2023; Heiran et al., 2022). This section will discuss the utilization of botanical drugs, herbal crude extracts, proprietary Chinese medicines, TCM formulas, and acupuncture for the management of the aforementioned conditions. Table 1 summarizes the primary clinical outcomes documented in TCM literature concerning the FGIDs and IBD management.

**TABLE 1:** Clinical trials of TCM intervention in the treatment of FGIDs and IBD.

Type	Disease	Name	n	Trial design	Intervention	Control	Treatment duration	Outcomes	Reference
FGIDs	FD	Biling Weitong Granules	240	Randomized, double-blind, multi-center, placebo-controlled, parallel group, stratified	Oral Biling Weitong Granules 5 g 3 times daily	Oral placebo 5 g 3 times daily	6 weeks	VAS	Wen et al. (2020)
		Jiawei Xiaoyao pill	144	Randomized, multi-center, placebo-controlled	Jiawei Xiaoyao pill, 12 g/d, 6 g 2 times daily	Placebo, 12 g/d, 6 g 2 times daily	4 weeks	GIS	Chen et al. (2020)
		Qi-Zhi-Wei-Tong granules	197	Randomized, double-blind, multi-center, placebo-controlled	Qi-Zhi-Wei-Tong granules, 2.5 g/bag, 2.5 g/time, 3 times daily	Placebo, 2.5 g/bag, 2.5 g/time, 3 times daily	4 weeks	Total effective rate of dyspeptic symptoms	Su et al. (2018)
		Xiang sha Liu jun zi granules	216	Randomized, double-blind, multi-center, placebo-controlled	Xiangsha Liujunzi Granules, 14 g/bag, 1 bag/time, 3 times daily	Placebo, 14 g/bag, 1 bag/time, 3 times daily	4 weeks	PDSS	Lv et al. (2017)
		Modified LiuJunZi decoction	160	Randomized, double-blind, placebo-controlled	Modified LiuJunZi decoction, 150 mL (50°C), 2 times daily	Placebo, 150 mL (50°C), 2 times daily	4 weeks	TDS, SDS^a^	Zhang et al. (2013)
		EA	712	Randomized, double-blind, multi-center, controlled	Group A: ST42, ST40, ST36, ST34; Group B: ST38, ST35, ST33, ST32; Group C: BL21, CV12; Group D: GB40, GB37, GB34, GB36; 0.5–1.5 mA, 2/100 Hz, 30 min	Group E: sham acupuncture of non-acupoints; Group F: itopride, 50 mg/time, 3 times daily	4 weeks	Nepean Dyspepsia Index, Symptom Index of Dyspepsia scale forms	Ma et al. (2012)
		Acupuncture	278	Randomized, triple-blind, multi-center, sham-controlled	Verum acupuncture: DU20, RN12, ST25, RN6, PC6, RN17, ST36, SP4 and individually selected acupoints: SP3, LR3, ST44; 20 min, 3 sessions weekly	Sham acupuncture, 20 min, 3 sessions weekly	4 weeks	The response rate, the elimination rate	Yang et al. (2020a)
	FD, IBS-D	EA	448	Randomized, prospective, parallel group controlled	He EA group, Shu-Mu EA group, He-Shu-Mu EA group: LI11, ST37, ST25, BL25; Intensity (until the nociceptive flexion reflex was achieved in a patient), 15 Hz	Loperamide group: oral loperamide 2 mg 3 times daily	4 weeks	Stool frequency	Zheng et al. (2016)
	IBS-D	Tong-Xie-Yao-Fang granules	160	Randomized, double-blind, placebo-controlled	Tong-Xie-Yao-Fang granules, 25.4 g/time, 3 times daily	Placebo, 25.4 g/time, 3 times daily	4 weeks	VAS	Chen et al. (2018)
			120	Randomized, controlled	Tong-Xie-Yao-Fang granule, 1 package/time, 2 times daily	Miyarisan, 2 tablet/time, 3 times daily	4 weeks	Bristol score	Pan et al. (2009)
			184		Tongxie Yaofang Granules, 2 doses daily	*Bifidobacterium lactobacillus* triple combination tablets, 500 mg/tablet, 2 tablet/time, 3 times daily	4 weeks	Traditional Chinese Medicine Syndrome Scoring, IBS-BSS, IBS-QOL	Zhang et al. (2023b)
		Personalized tongxie formula	1,044	Randomized, blinded, multi-center, placebo-controlled	Personalized tongxie, warm decoction, 3 times daily	Pinaverium 50 mg/tablet, 3 times daily; placebo, 3 times daily	4 weeks	Abdominal pain, Bristol stool form scale	Fan et al. (2017)
		Chang’an Ⅰ Recipe	216	Randomized, double-blind, multi-center, placebo-controlled	Chang’an Ⅰ Recipe, 150 mL/bag, 1 bag/time, 3 times daily	CM placebo, 150 mL/bag, 1 bag/time, 3 times daily	8 weeks	IBS-SSS, AR	Tang et al. (2018)
		Yigan Fupi Decoction	122	Randomized, controlled	Yigan Fupi Decoction, 1 dose daily, divided into 2 doses	Pinaverium Bromide Tablet, 50 mg/pill, 1pill/time, 3 times daily	4 weeks	IBS-BSS	Chen et al. (2014)
		Tiaohe Ganpi Hexin Decoction	40	Randomized, controlled	Oral Tiaohe Ganpi Hexin Decoction 1 time daily	Oral pinaverium 50mg, 3 times daily	4 weeks	TCM syndrome score, total obviously effective rate, disappearance rate of symptoms, and clinical symptom score	Liang et al. (2009)
		Mild moxibustion	76	Randomized, controlled	ST25, ST36; 43°C ± 1 °C, 30 min, 3 times weekly	ST25, ST36; 37°C ± 1 °C, 30 min, 3 times weekly	6 weeks	The global treatment effect questionnaire, IBS-SSS, IBS-QOL	Wang et al. (2022c)
		Acupuncture	81	Randomized, controlled	Regulating mind and spleen: GV20, GV29, ST25, ST36, ST37, SP6, LR3; 30 min, once every other day, 3 times weekly	Oral Pinaverium bromide tablet, 50 mg/time, 3 times daily	6 weeks	IBS-SSS, PSQI	Li et al. (2017)
	IBS-D, IBS-C	Acupuncture	519 (IBS-D n = 382, IBS-C n = 137)	Randomized, multi-center, controlled	Tiaoshen Jianpi: GV20, GV29, ST25, ST37, ST36, SP6, LR3; 30 min, once every other day, 3 times weekly	Pinaverium group: 50 mg/pill, 50 mg/time, 3 time/d Polyethylene glycol group: 10 g/pill, 20 g/time, 1 time daily	6 weeks	IBS-SSS IBS-QOL	Guo et al. (2021a)
	IBS	Dinggui Oil Capsule	198	Randomized, double-blind, placebo-controlled	High-dose Dinggui Oil group (DGO-H): 1.2 g, 3 times daily Low-dose Dinggui Oil group (DGO-L): 0.8 g, 3 times daily	Placebo group (placebo): 5.0 g, 3 times daily	2 weeks	0–10 numeric pain intensity scale, laboratory inspection indicators	Zhang et al. (2007)
		Acupuncture	531	Randomized, multi-center, controlled	GV20, GV29, LR3, ST36, SP6, ST25, ST37; 30 min, once every other day, 3 times weekly	PEG 4000 group: oral PEG 4000 powder 10 g, 2 sachets daily Pinaverium Bromide group: oral pinaverium bromide tablets 50 mg tablet, 3 times daily	6 weeks	IBS-SSS, IBS-QOL	Pei et al. (2020)
IBD	UC	Indigo naturalis	86	Randomized, double-blind, multi-center	Indigo naturalis 250 mg, 125 mg, 62.5 mg. Respectively 4 pill/time, 2 times daily	Placebo, 4 pill/time, 2 times daily	8 weeks	Mayo score	Naganuma et al. (2018)
		Composite Sophora Colon-soluble Capsules	160	Randomized, double-blind, multi-center, placebo-controlled	Oral Composite Sophora Colon-soluble Capsules 0.4 g/pill, 4 pill/time, 3 times daily and Etiasa simulated placebo 1 g/time, 4 times daily	Oral Etiasa 1 g/time, 4 times daily and Composite Sophora Colon-soluble Capsule simulated placebo 4 pill/time, 3 times daily	8 weeks	Comprehensive therapeutic efficacy, effects on syndrome, safety of treatment, changes of endoscopic features, Chinese medical syndrome scores and symptom score, AI of UC, microscopic pathology	Tong et al. (2011)
		A. Paniculata ethanol extract (HMPL-004)	224	Randomized, double-blind, multi-center, placebo-controlled	Oral capsules containing A. paniculata ethanol extract (HMPL-004) at doses of 1,200 mg or 1800 mg, administered in three divided doses	Oral placebo, 3 times daily	8 weeks	Mayo Score	Sandborn et al. (2013)
			120	Randomized, double-blind, multi-center	HMPL-004 400 mg t.d.s., 1,200 mg daily	Mesalazine SR Granules 1,500 mg t.d.s., 4,500 mg daily	8 weeks	General evaluation, clinical evaluation	Tang et al. (2011)
		Fufangkushen colon-coated capsule	320	Randomized, double-blind, multi-center, double-dummy, controlled	FCC plus HD placebo treatment (at advised dosage)	HD plus FCC placebo (at advised dosage)	8 weeks	Mayo score	Gong et al. (2012)
		Qingchang Huashi Recipe	60	Randomized, controlled	Qingchang Huashi Recipe, 1 dose each time, decocted twice, mixed to 300 mL, taken in two portions; combined with enema of guanchang recipe, decocted twice, mixed and concentrated to 120 mL, applied before sleep every evening, with an interval of 12 days after 12 successive days	Mesalazine Enteric-coated Tablet 0.25 g/tablet, 1 g/time, 4 times daily	8 weeks	The symptom integral, the colonoscopic results, the pathological efficacy, the remission rate	He et al. (2012)
	CD	Boswellia serrata extract (Boswelan)	108	Randomized, double-blind, multi-center, placebo-controlled	Boswelan 3 × 2 capsules/day, 400 mg each	Placebo, 2 capsules/time, 3times daily	52 weeks	CDAI	Holtmeier et al. (2011)
		Tripterygium wilfordii Hook F	137	Randomized, controlled, open-label	Low-dose TwHF, 1.5 mg/kg/d High-dose TwHF 2.0 mg/kg/d	Mesalazine, 3 g/d	52 weeks	CDAI	Sun et al. (2015)
		Wormwood	20	Randomized, open label, multi-center	Seda-Crohn^R^ capsule, 250 mg/capsule, 3 capsule/time, 3 times daily	Placebo	6 weeks	HAMD	Krebs et al. (2010)
		Acupuncture and moxibustion	66	Randomized, sham controlled, parallel-group	Acupuncture: CV12, ST37, SP6, SP4, LR3, KI3, LI4, LI11; moxibustion: ST36, ST25, 43°C ± 1°C; 30 min, 3 sessions weekly	Sham acupuncture	12 weeks	CDAI	Bao et al. (2022)

AI, activity index; AR, adequate relief; CD, Crohn’s disease; CDAI, Crohn’s disease activity index; CM, chinese medicine; EA, electroacupuncture; FCC, fufangkushen colon-coated capsule; FD, functional dyspepsia; FGIDs, functional gastrointestinal disorders; GIS, gastrointestinal symptom score; HAMD, Hamilton’s Depression Scale; HD, huidi, mesalazine enteric-coated tablets; IBD, inflammatory bowel disease; IBS, irritable bowel syndrome; IBS-BSS, IBS, bowel symptom severity scale; IBS-C, constipation-predominant irritable bowel syndrome; IBS-D, diarrhea predominant-irritable bowel syndrome; IBS-QOL, irritable bowel syndrome Quality of Life; IBS-SSS, irritable bowel syndrome Symptom Severity Score; PDSS, postprandial discomfort severity scale; PSQI, pittsburgh sleep quality index; SDS, single dyspepsia symptom scale; TCM, traditional Chinese medicine; TDS, total dyspepsia symptom scale; UC, ulcerative colitis; VAS, visual analog score.

### 4.1 Clinical efficacy and safety of TCM in FGIDs

Clinical studies evaluating the efficacy of TCM interventions for FGIDs have used several efficacy evaluation scales and questionnaires, of which the IBS-Symptom Severity Score and the Bristol Stool Scale are the main tools used to assess efficacy in IBS, and the Single Dyspepsia Symptom Scale is the main tool used to assess efficacy in FD. Xiangsha Liujunzi granules (14 g three times daily) relieved FD symptoms and reduced recurrence for up to 4 weeks post-treatment (Lv et al., 2017). Tong-Xie-Yao-Fang (TXYF) granules are effective in treating IBS-D in multiple clinical trials. In a multicenter randomized clinical trial with a total sample of 1044 patients, personalized TXYF (warm decoction) for IBS-D was significantly superior to pivacurium bromide in improving fecal characteristics (Fan et al., 2017). The results of another randomized clinical trial with 12 weeks of treatment showed that patients receiving TXYF (25.4 g three times daily) had significantly lower abdominal pain visual analog scale scores than patients who received the placebo at 3–7 weeks of treatment (Chen et al., 2018). The number of mast cells in the colonic mucosa was significantly reduced after TXYF treatment and was significantly different between the pretreatment and control groups (Pan et al., 2009).

Acupuncture has demonstrated efficacy in treating various IBS subtypes, predominantly IBS-D. Recent systematic reviews have confirmed the effectiveness of acupuncture in alleviating abdominal pain symptoms in patients with IBS (Yang et al., 2022) and have highlighted its safety profile for pediatric and adult populations (Cai et al., 2024). Key acupoints such as “Tianshu” (ST25), “Zusanli” (ST36), and “Shangjuxu” (ST37) are commonly targeted in acupuncture interventions. A study indicated that acupuncture, particularly when targeting mental and spleen regulation, outperformed pivacurium bromide in the early-stage relief of abdominal pain in patients with IBS-D (Li et al., 2017). Furthermore, acupuncture has shown promise in modulating constipation and diarrhea in individuals with IBS (Guo J. et al., 2021). In FD, acupuncture is effective in ameliorating symptoms of postprandial distress syndrome, including postprandial fullness, early satiety, and epigastric distension, with therapeutic benefits persisting for up to 8 weeks post-treatment cessation (Yang J. W. et al., 2020). A 4-week electroacupuncture treatment of 333 patients with IBS-D or FD improved patients’ quality of life, reduced defecation frequency, and improve stool consistency (Zheng et al., 2016).

TCM treatments for FGIDs are generally considered safe when administered by trained practitioners. However, potential side effects can occur, including allergic reactions to herbs, gastrointestinal upset, and in rare cases, interactions with conventional medications.

### 4.2 Clinical efficacy and safety of TCM in IBD

The Mayo score and Crohn’s Disease Activity Index are commonly utilized efficacy assessment tools in clinical studies evaluating TCM interventions for IBD. *Andrographis paniculata* ethanol extract and Qing-Chang-Hua-Shi (QCHS) granules are frequently employed for UC, whereas boswelan is commonly used for CD. In an 8-week randomized clinical trial involving patients with UC, *Andrographis paniculata* ethanol extract at a dose of 400 mg three times daily was superior to mesalazine at a dose of 1,500 mg three times daily (Tang et al., 2011) and to placebo at 1800 mg twice daily (Sandborn et al., 2013). However, some adverse effects of the ethanol extract of *Andrographis paniculata*, such as fever, rash, and high C-reactive protein levels, were reported in a previous clinical trial (Tang et al., 2011).These side effects may be attributed to the immunostimulatory properties of the extract, which can cause an exaggerated immune response in some individuals.As such, caution should be taken when utilizing it in clinical practice. Boswelan has shown superiority over placebo in symptom relief and relapse prevention in patients with CD (Holtmeier et al., 2011). A study indicated that QCHS (150 mL twice daily) was more effective in improving UC symptoms and has a better safety profile than the 0.25 g/tablet, 1 g/time, four times daily dose of mesalazine (He et al., 2012).

Acupuncture and moxibustion are commonly employed to treat IBD, which are frequently targeted acupoints such as “Tianshu” (ST25) and “Zusanli” (ST36). In patients with UC, spaced moxibustion has shown significant efficacy in alleviating symptoms and reducing the expression of inflammatory markers (Qi et al., 2021; Zhou et al., 2009). Furthermore, acupuncture and moxibustion lead to notable improvements in symptomatology and decreased expression of relevant inflammatory markers in patients with IBD. Specifically, studies have highlighted the safety and efficacy of acupuncture and moxibustion at acupoints “Zhongwan” (CV12), “Shangjuxu” (ST37), and “Tianshu” (ST25) in individuals with mild-to-moderate active CD who exhibit poor responsiveness to or intolerance of conventional medications (Bao et al., 2022). These findings have been recognized as one of the top 10 Academic Advances in Traditional Chinese Medicine for 2022.

In recent years, peppermint and TXYF have been incorporated into guidelines and expert consensus for IBS (Disorders et al., 2020; Lacy et al., 2021). For patients with mild-to-moderate active UC, the 2022 Chinese expert consensus on the diagnosis and treatment of FD strongly recommends the combined use of QCHS and herbal enema (Group et al., 2024). The Chinese consensus on FD (Group et al., 2022) suggests the use of TCM such as Xiangsha Liujunzi granules and Biling Weitong granules, along with acupoint stimulation. TCM and acupoint stimulation are considered safe and effective in managing FGIDs and IBD in clinical settings.

The safety profile of TCM in IBD is generally favorable, but care must be taken to avoid herb-drug interactions, especially with immunosuppressive therapies. Potential adverse effects include gastrointestinal disturbances and allergic reactions. The quality control of herbal products is critical to ensure safety.

TCM offers promising complementary and alternative treatment options for FGIDs and IBD, with potential benefits in symptom relief and quality of life improvement. However, patients should seek treatment from qualified TCM practitioners and consider TCM as part of an integrated treatment plan. Ongoing research and well-designed clinical trials are essential to further validate the efficacy and safety of TCM in these conditions.

## 5 Potential action mechanisms of TCM in FGIDs and IBD

TCM shows promise in treating FGIDs and IBD; nonetheless, the specific mechanism remains undefined. A possible explanation for the effectiveness of TCM in these conditions is its ability to regulate the gut microbiome, which is crucial in the development and progression of FGIDs and IBD. TCM botanical drugs and formulations have antimicrobial and anti-inflammatory properties, which may help restore balance to the gut microbiome and alleviate symptoms. In addition, TCM often takes a holistic approach, addressing not only physical symptoms but also contributing emotional and psychological factors. This comprehensive approach may also contribute to the success of TCM in treating FGIDs and IBD. However, more studies are needed to fully understand the mechanism of action of TCM in these conditions and optimize its use in clinical practice. This section summarized the major research conducted in the last 5 years regarding how TCM affects FGIDs and IBD.

### 5.1 Modulation of intestinal immune and inflammatory functions

#### 5.1.1 FGIDs

The dried mycorrhizae of *Poria cocos* (Schw.) Wolf of family Polyporaceae are used as medicinal components of *P. cocos*, which are commonly used in the treatment of diarrhea and other diseases because of its ability to strengthen the spleen and stomach and regulate water metabolism (Chinese Pharmacopoeia Commission, 2020). A 2-weeks study on three extracts of *P. cocos* (triterpenoid, water-soluble polysaccharide, and acidic polysaccharide) showed that water-soluble (7.5 g/kg) and acidic (1.0 g/kg) polysaccharides could help regulate Th1/Th2 and Th17/Treg homeostasis in alternate-day fasting and weight-loaded forced swimming-induced FD rats. Triterpenoid (7.5 g/kg) can promote repair of the gastrointestinal mucosa in rats, and Buzhongyiqi pill (4.5 g/kg) was used as the positive control (Tu et al., 2022). Baicalin–berberine nanoparticles, a combination of baicalin extracts from *Scutellaria baicalensis* Georgi and berberine extracts from *Coptis chinensis* Franch., reduced the expression of nuclear factor-kappa B (NF-κB) in the colonic tissues of IBS-D mice and decreased basophil granulocyte and leukomonocyte levels in the whole blood of mice for 10 days (Li L. et al., 2020). Wei-Tong-Xin is a Chinese herbal formula composed of five botanical drugs, namely, *Rheum pal matum* L., *Aucklandia lappa* Decne, *Gleditsia sinensis* Lam, *Pharbitis nil* (L.) Choisy, and *Glycyrrhiza uralensis* Fisch. Zhang X. et al. (2022) treated FD rats with Wei-Tong-Xin (0.5, 1.0, and 2.0 g/kg) for 3 days at different administration doses, which significantly promoted gastrointestinal motility in a dose-dependent manner. The therapeutic effect may be accomplished by downregulating the TLR4/myeloid differentiation factor 88 signaling pathway and reducing the expression of glucagon-like peptide 1 and its receptor. Electroacupuncture at “Zusanli” (ST36) was reported to reduce the number of mast cells, inhibit their degranulation release in FD rats, and downregulate the expression of nerve growth factor and its receptor (Dong et al., 2022). It can also reduce the amount of TLR4 in colon tissues to reduce the release of proinflammatory factors from mast cell degranulation and restore the normal role of the intestinal mucosal barrier (Yang et al., 2019). Chang’an II (2.85, 5.71, and 11.42 g/kg), derived from TXYF (Wang et al., 2015), was used to treat 2,4,6-trinitrobenzene sulfonic acid-induced post-infectious IBS model rats for 2 weeks. It reduced inflammation in post-infectious IBS model rats by increasing the ratio of CD4^+^/CD8^+^ cells in the lamina propria and submucosa of the small intestinal mucosa and decreasing the levels of IL-1β and IL-4.

#### 5.1.2 IBD

To regulate the innate immune system, the mechanism of TCM mostly involves TLR4-related inflammatory pathways and macrophage polarization. For example, *G*. *uralensis* Fisch. (Shi et al., 2022), *Portulaca oleracea* L. polysaccharide (Yang et al., 2023), and others increase the expression of intestinal tight junction proteins to repair the intestinal barrier by regulating the downregulation of the TLR4-myeloid differentiation factor 88-NF-κB pathway. Pingwei San (Zhang et al., 2019), baicalin and emodin coadministration (Xu B. et al., 2021), and honokiol (Wang N. et al., 2022) inhibited downstream NF-κB activation through TLR4 inhibition and peroxisome proliferator-activated receptor-γ activation. A 10-day study in DSS-induced UC mice showed that coptisine (25, 50, and 100 mg/kg) inhibited the MAPK/extracellular signal-regulated kinase signaling pathway and increased N6-methyladenosine RNA methylation to regulate macrophage polarization.5-ASA (200 mg/kg) was used as the positive control (Zhao et al., 2024), and adenosine 5′-monophosphate-activated protein kinase, a polarization regulator, was activated in the peritoneal macrophages of mice with DSS-induced colitis by platycodon D at a dose of 10 mg/kg (Guo R. et al., 2021). TXYF (5.6 and 11.2 g/kg) inhibited NF-κB activation and thus reduced NLRP3 gene expression, the optimal dose is 11.2 g/kg, and a positive control was not set (Zhang H. Y. et al., 2022). Together, these three factors reduce M1 polarization, increase M2 polarization, and reduce diarrhea in model animals. In a 1-week study of 2,4,6-trinitrobenzene sulfonic acid-induced IBD mice treated with electroacupuncture stimulation at bilateral “Dachangshu” (BL25), electroacupuncture inhibited the activation of macrophages in the colonic mucosa and reduced the release of inflammatory factors through the activation of cannabinoid CB2 receptors (Zhang H. et al., 2022). Qinghua Quyu Jianpi decoction (14.17 g/kg) can activate the Wnt pathway, promoting epithelial cell turnover, reducing apoptosis, activating the Wnt pathway by inducing nuclear translocation of β-catenin, accelerating the cell cycle, and promoting cell proliferation (Qu et al., 2023).

To regulate the adaptive immune system, studies on the anti-inflammatory effects of TCM have focused on restoring the Th17/Treg cell balance in IBD, i.e., promoting the conversion of naive CD4^+^ T cells to Tregs. For example, paeoniflorin (Zheng et al., 2020), Kuijie decoction (Peng et al., 2024), classic formula Yiyi Fuzi Baijiang formula (Liu et al., 2023d), The Gegen Qinlian decoction, a TCM prescription documented in an ancient text dating back approximately 1800 years ago, are commonly used for the treatment of damp–heat diarrhea, and Gegen Qinlian decoction (1.5 and 7.5 g/kg) was found to reduce the Th17-like transformation of Tregs and attenuate the intestinal immune response of DSS-induced mice by inhibiting the overactivation of signal transducers and activators of transcription 3 (Zhao et al., 2021). After 1 week trial, Astragaloside IV (50 and 100 mg/kg) inhibited the overactivation of the Notch signaling pathway in a dose-dependent manner to restore the normal Th17/Treg cell ratio, decrease IL-17A and IL-21 levels, increase body weight, and decrease the disease activity index in DSS-induced colitis mice (Zhong et al., 2022a) (Table 2).

**TABLE 2:** Mechanism of intestinal immune and inflammatory functions in TCM intervention of FGIDs and IBD.

Type	Disease	Intervention	Main Ingredients/Acupoints	Experiment models	Mechanisms	Reference
FGIDs	FD	Triterpenoid, water-soluble polysaccharide and acidic polysaccharide	Triterpenoid; water-soluble polysaccharide; acidic polysaccharide	Alternate-day fasting and WLFS-induced FD rats	Promoting the repair of gastrointestinal mucosa; regulating the balance between the Th1/Th2 axis and the Th17/Treg axis	Tu et al. (2022)
					Regulating brain-gut peptides more effectively, enhancing immunity	
					Enhancing immunity via the TLR and JNK signaling pathways	
		Wei-Tong-Xin (WTX)	Catechin, chlorogenic acid, liquiritin, aloe-emodin, etc.	CIS-induced FD rats	Inactivating the TLR4/MyD88 signaling pathway to inhibit the occurrence of gastric antral inflammation	Zhang et al. (2022c)
					Reversing the inhibitory effect of GLP-1 on gastric motility	
		EA	ST36	Iodoacetamide combined with rat tail clamping method-induced rats	Inhibiting duodenal mast cells	Dong et al. (2022)
					Regulating the expressions of NGF and its receptor to improve the low-grade inflammatory response of duodenum	
				Chronic visceral hyper-Sensitivity rats	Ameliorating visceral hypersensitivity via decreasing the level of pro-inflammatory cytokines; down regulating TLR4 expression, decreasing the release of mast cell	Yang et al. (2019)
	IBS-D	Berberine-Based Chinese medicine assembled nanostructures	Berberine and baicalin	Chronic restraint stress plus Senna alexandrina Mill decoction-induced IBS-D mice	Reducing the levels of 5-HT, VIP, and CHAT in colon tissues or of serum; reducing the expressions of NF-kB in colon tissues and changed the levels of BASO and LYMPH in whole blood	Li et al. (2020c)
					Altering intestinal flora of Bacteroidia, Deferribacteres, Verrucomicrobia, Candidatus_Saccharibacteria, and Cyanobacteria	
	IBS	Chang’an II	Paeoniflorin, hesperidin, atractylenolide, etc.	TNBS-induced PI-IBS rats	Increasing CD4^+^/CD8^+^ cell ratio in lamina propria and submucosa, reducing IL-1β and increasing IL-4 expression in intestinal mucosa	Wang et al. (2015)
IBD	UC	Berberine	Berberine	DSS-induced experimental colitis mice	Enhancing the population and residence of EGCs and regulating the enteric glial-immune-epithelial cells interactions	Li et al. (2020b)
		Qinghua Quyu Jianpi Decoction (QQJD)	Beta-sitosterol, sitosterol, Spinasterol, etc.	DSS-induced UC mice	Activating the Wnt pathway to promote epithelial cell renewal, reducing apoptosis, and repairing the mucosal barrier; activating the Wnt pathway by inducing nuclear translocation of β-catenin, accelerating the cell cycle and promoting cell proliferation	Qu et al. (2023)
		Sargentodoxa cuneata	Protocatechuic acid, Vanillic acid glucoside, Hydroxytyrosol glucoside, etc.	DSS-induced UC mice	Preserving the integrity of the epithelial and mucosal barrier; suppressing the mRNA expression of pro-inflammatory cytokines by impeding intestinal epithelial necroptosis	Wang et al. (2024b)
		Astragalus polysaccharide	Astragalus polysaccharide	DSS-induced colitis mice	Regulating the balance of Tfh/Treg cells	Zhong et al. (2022b)
		Astragaloside Ⅳ	Astragaloside Ⅳ	DSS-induced colitis mice	Regulating the balance of Th17/Treg cells	Zhong et al. (2022a)
		Baicalin	Baicalin	TNBS-induced UC rats		Zhu et al. (2020)
		Paeoniflorin (PF)	Paeoniflorin (PF)	TNBS-induced colitis mice		Zheng et al. (2020)
		Compound sophorae decoction	Matrine, Oxymatrine, Liquiritin, etc.	DSS-induced colitis mice		Xu et al. (2019)
		Kuijie decoction (KJD)	Gallic acid, Kaempferol, Rutin, Isoquercitrin, Genistein, Quercetin, Hesperidin, Benzaldehyde, Oleanolic aldehyde, Isovaleric acid, Riboflavin, Phloretin, etc.	DSS-induced UC mice		Peng et al. (2024)
		Kuijieling (KJL)	Mairin, 12-senecioyl-2E,8E,10E-atractylentriol, paeoniflorgenone, Inermine, etc.	TNBS-induced UC rats		Xiao et al. (2024)
		Yiyi Fuzi Baijiang formula (YFB)	Benzoylmesaconitine, Benzoylaconitine, Benzoylhypaconitine, Mesaconitine, Hypaconitine, Aconitine	TNBS-induced UC rats		Liu et al. (2023d)
		Curcumin	Curcumin	DSS-induced UC in DB mice	Improving the composition of gut microbiota	Xiao et al. (2022)
		Sophora flavescens Aiton total flavonoids extracts (SFE)	Kurarinone	UC mice and abnormal immune RAW 264.7 cell models	Regulating the balance of Th17/Treg cell differentiation	Li et al. (2022c)
					Down regulating the JAK2/STAT3 signaling pathway	
		Gegen Qinlian decoction (GQD)	Berberine, baicalin, and puerarin, etc.	DSS-induced mice	Suppressing IL-6/JAK2/STAT3 signaling to restore Treg and Th17 cell homeostasis	Zhao et al. (2021)
		Paris polyphylla Sm. (P.P)	Polyphyllin VI	DSS-induced acute colitis mice	Restoring the Treg/Th17 balance via the PPAR-γ/STAT3/HIF-1α axis	He et al. (2023)
		Jiaoqi powder (JQP)	Quercetin, Stigmasterol, Maltol, Lysine, etc.	DSS-induced colitis C57BL/6 mice	Regulating the Th17/Treg cell balance	Wen et al. (2021)
					Impairing DSS’s ability to induce high expression levels of NF-κB/p65, IL-1β, IL-6, and TNF-α; reducing the levels of COX-2, CCL2, CXCL2, HIF-1α, MMP3 and MMP9	
		Celastrol (CSR)	Celastrol (CSR)	DSS-induced colitis mice	Improving the balances of Treg/Th1 and Treg/Th17	Li et al. (2022a)
					Down regulating the production of pro-inflammatory cytokines, up-regulating the number of anti-inflammatory mediators at both mRNA and protein levels	
		Costunolide	Costunolide	DSS-induced UC mice	Inhibiting HIF1α/glycolysis-mediated Th17 differentiation	Lv et al. (2021)
		Cinnamon	Cinnamaldehyde (CA)	DSS-induced colitis	Suppressing Th17 cells via S1P2 pathway	Qu et al. (2021)
					Regulating lncRNA H19 and MIAT	
		Cryptotanshinone (CTS)	Cryptotanshinone (CTS)	DSS-induced chronic colitis mice	Suppressing Th17 cell differentiation	Fan et al. (2022)
					Suppressing STAT3 activation	
		Qing-Chang-Hua-Shi granule (QCHS)	Berberine, Baicalin, Coumarin, Ferulic acid, Paeoniflorin, etc.	DSS-induced mice	Driving Th17 cell differentiation by activating NLRP6 inflammasome pathway	Cheng et al. (2022a)
					Regulating the maturation of IL-1β and IL-18 to affect inflammation	
		Xianglian pill (XLP)	Coptisine, Palmatine, Berberine, Quercetin, etc.	DSS-induced UC mice	Inhibiting Th17 cell differentiation by suppressing the JAK2-STAT3 pathway	Liu et al. (2021)
				DSS-induced UC rats	Inducing the differentiation of Treg possibly by the regulation of the PHD2/HIF-1α pathway via decreasing microbial succinate production	Liu et al. (2023b)
				DSS-induced UC mice	Regulating the TLR4/MyD88/NF-κB signaling pathway	Dai et al. (2023)
				DSS-induced colitis mice; BMDMs, PBMCs, RAW264.7 and THP-1 cells	Regulating macrophage alternative activation via tipping the balance of STAT1/PPARγ	Zhou et al. (2023a)
		Chimonanthus nitens Oliv. leaf granule (COG)	Scopoletin, Isofraxidin, Scoparone, Rutin, Chimonanthine, Calycanthine	DSS-Induced colitis mice	Inhibiting immune-inflammatory responses and oxidative stress; regulating mTreg cell responses	Huang et al. (2022a)
		Glycyrrhiza uralensis Fisch	Liquiritin, glycyrrhizic acid	DSS-induced BALB/c rats	Affecting the protein expression of TLR4/MyD88/NF-κB	Shi et al. (2022)
					Altering the imbalance of Th-cell differentiation	
		β-patchoulene (β-PAE)	β-patchoulene (β-PAE)	DSS-induced UC mice	Suppressing TLR4/MyD88/NF-κB and ROCK1/MLC2 signaling pathway	Liu et al. (2020c)
		Oxyberberine (OBB)	Oxyberberine (OBB)	DSS-induced acute colitis mice	Regulating the TLR4/MyD88/NF-κB signaling pathway	Li et al. (2020a)
		Portulaca oleracea L. polysaccharide (POL-P)	Mannose, Rhamnose, Glucuronic acid, Galacturonic acid, Glucose, Galactose, and Arabinose	DSS-induced colitis mice		Yang et al. (2023)
		Sanhuang Xiexin decoction (SXD)	Baicalin, Berberine, Wogonoside, etc.	DSS-induced UC mice	Inhibiting the expression levels of p-IκBα, TLR4, MyD88, and p65	Wu et al. (2022)
					Inhibiting the expression of inflammatory cytokines and oxidative stress indicators	
		Anemoside B4	Anemoside B4	DSS-induced colitis rats	Inhibiting the activation of the TLR4 signaling pathway, down regulating the expression of key proteins in the TLR4/NF-κB/MAPK signaling pathway	Ma et al. (2020)
		Methyl gallate (MG)	Methyl gallate (MG)	DSS-induced UC mice	Regulating the TLR4/NF-κB pathway	Zhou et al. (2022)
		Vitexin	Vitexin	DSS-induced colitis mice		Duan et al. (2020)
		Honokiol	Honokiol	DSS-induced UC rats	Activating the PPAR-γ–TLR4–NF-κB signaling pathway	Wang et al. (2022b)
					Inhibiting gasdermin-D-mediated cell pyroptosis	
		Baicalin and emodin	Baicalin and emodin	DSS-induced colitis rats	Decreasing the expression of CD14/TLR4/NF-κB pathway proteins	Xu et al. (2021a)
					Increasing the expression of PPAR-γ protein	
		Ping weisan (PWS)	Liquiritin, Hesperidin, Isoglycyrrhizin, Atractylenolide III, Glycyrrhizic acid	DSS-induced colitis mice	Reducing TNF-α, IL-1β and IL-12 production	Zhang et al. (2019)
					Suppressing NF-κB pathway activation by regulating the expression of TLR4 and PPARγ	
		Ethanol extract of Piper wallichii (EEPW)	Isofutoquinol A, hancinone C, futoquinol, etc.	DSS-induced UC in BALB/c mice	Inhibiting protein expression of TLR4, p-IκB-α, p-p65, and COX-2; affecting TLR4/NF-κB/COX-2 signaling pathway	Zhao et al. (2023a)
		Clinopodium chinense Kuntze (CC)	Triterpene and phenolic compounds	LPS-induced RAW 264.7cells and DSS-induced UC rats	Inhibiting inflammation by LPS-TLR4-NF-κB-iNOS/COX-2 signaling pathway	Wang et al. (2023b)
					Restoring the abnormal endogenous metabolite levels	
		Schisandra chinensis (Turcz.) Baill. extract (SCE)	Dibenzocyclooctene lignans	DSS-induced colitis mice	Regulating the TLR4/NF-κB/NLRP3 inflammasome pathway	Bian et al. (2022)
					Reversing the GM imbalance	
		Ganluyin (GLY)	Naringin, neohesperidin, baicalin, wogonoside	DSS-induced UC mice	Improving the colonic mucosal barrier	Xiong et al. (2022)
					Inhibiting the enteric-origin LPS/TLR4/NF-κB inflammatory pathway	
					Increasing the expression of the tight junction proteins, occludin, claudin-1, and ZO-1	
		Angelica oil (AO)	Ligustilide, linoleic acid	DSS-induced UC rats	Enhancing the expression of TJs	Liu et al. (2023a)
					Inhibiting the activation of the S100A8/A9/TLR4 signaling pathway	
		Phillygenin (PHI)	Phillygenin (PHI)	DSS-induced colitis mice	Inhibiting the activation of tyrosine kinase Src mediated by TLR4, then reducing the phosphorylation of downstream proteins p38, JNK, and NF-κB	Xue et al. (2023)
					Protecting goblet cells, promoting gene expressions of Clca1, Slc26a3, and Aqp8, reducing epithelial cell apoptosis, reversing the levels of oxidative stress (MPO, SOD, and MDA) and inflammatory cytokines (TNF-α, IL-1β, IL-6, and IL-10)	
					Increasing TJs: ZO-1 and occludin	
		20(S)-Protopanaxadiol saponins (PDS)	20(S)-Protopanaxadiol saponins (PDS)	DSS-induced UC mice	Blocking the binding of HMGB1 to TLR4	Chen et al. (2023)
		Ulva pertusa	Rhamnose, aspartic acid, lipid, etc.	DNBS-induced colitis mice	Modulating innate and adaptative immune-inflammatory responses; modulating TLR4 and NLRP3 inflammasome	Ardizzone et al. (2023)
		Oxymatrine (OMT)	Oxymatrine (OMT)	DSS-induced colitis mice	Inhibiting the TLR/NF-κB signaling pathway	Liu et al. (2023c)
					Regulating the crosstalk of inflammatory DCs and GM	
		Wu-Mei-Wan (WMW)	Citric acid, Phellodendrine, ferulic acid, Coptisine, Jatrorrhizine, etc.	DSS-induced colitis mice	Inhibiting the Notch/NF-κB/NLRP3 pathway to inhibit the activation of colonic macrophages	Yan et al. (2022)
					Promoting the proliferation of colonic stem cells by regulation of the Hippo/YAP signaling pathway	
		Coptisine (COP)	Coptisine (COP)	DSS-induced UC mice	Increasing the expression of METTL14, which enhanced m6A methylation and ultimately improved the stability of TSC1 mRNA	Zhao et al. (2024)
					Regulating the polarization of macrophages	
		Platycodin D (PLD)	Platycodin D (PLD)	DSS-induced colitis mice and LPS-induced inflammation mice	Increasing and protecting intestinal barrier protein: ZO-1, occludin and claudin-1	Guo et al. (2021b)
					Regulating macrophage polarization via activation of AMPK	
		Compound sophorae decoction (CSD)	Matrine, Oxymatrine, Gallic acid, Liquiritin, Glycyrrhizic acid, Ginsenoside Rb1, Notoginsenoside R1, Indigo, Indirubin	DSS-induced colitis mice	Regulating notch signaling, decreasing the ratio of M1/M2	Wu et al. (2021)
					Up regulating the expression of ZO-1 and occludin	
					Up regulating the secretion of MUC2	
		Tongxie-Yaofang formula (TXYF)	Sitosterol, 12-senecioyl-2E,8E,10E-atractylentriol, beta-sitosterol, benzoyl paeoniflorin, etc.	DSS-induced colitis mice; LPS, IFN- and ATP-induced BMDM cell	Interfering with macrophage infiltration and polarization by repressing NF-κB/NLRP3 signaling pathway activation	Zhang et al. (2022b)
		Fructus ligustri lucidi (FLL)	Kaempferol, lueolin	DSS-induced colitis mice	Reducing the transition of mφs to the proinflammatory phenotype, promoting Mφs-regulated wound healing	Yu et al. (2022b)
					Suppressing the production of ROS in IOs and crypts	
		Cinnamaldehyde (CA)	Cinnamaldehyde (CA)	DSS-induced colitis mice	Inhibiting NLRP3 inflammasome activation and miR-21 and miR-155 levels in colons and macrophage	Qu et al. (2019)
		Geniposide	Geniposide	DSS-induced acute colitis mice; LPS-induced BMDM cell or RAW264.7 cell models	Suppressing NLRP3 inflammasome in macrophages by AMPK/Sirt1-dependent signaling	Pu et al. (2020)
		American ginseng (AG)	Panaxynol (PA)	DSS-induced colitis mice	Activating the Nrf2 pathway and decreasing the oxidative stress in mΦs and colon epithelial cells	Chaparala et al. (2020)
		Wei Chang An pill (WCA)	Cynaropicrin, isorhamnetin, eucalyptol, etc.	TNBS-induced UC rats; LPS-induced monocyte THP-1 cells	Inhibiting inflammation-induced EMT progression	Qi et al. (2023)
		EA	ST36	DSS-induced colitis rats	CXCL1 is the target of EA, underlying immune mechanism related to Th1 cytokine IFN-γ	Zhang et al. (2023a)
	CD	Xue-Jie-San (XJS)	Daidzein, Loureirin B, Dehydrocostus lactone, Atractylenolide I	TNBS-induced colitis rats	Restraining ferroptosis in IECs to ameliorate experimental colitis by inhibition of FGL1/NF-κB/STAT3 positive feedback loop	Gao et al. (2023)
		Brucea javanica oil emulsion (BJOE)	Oleic acid, linoleic acid	TNBS-induced CD rats	Suppressing TLR4-linked NF-κB signal pathway and down-regulating inflammatory mediators	Huang et al. (2019)
	IBD	Indirubin (IDRB)	Indirubin (IDRB)	DSS-induced colitis mice	Regulating the differentiation of T cells by mediating the maturation of BMDCs through αVβ8	Zhang et al. (2023c)
		EA	BL25	TNBS-induced IBD mice	Activating CB2 receptors and subsequent inhibition of macrophage activation and expression of IL-1β and iNOS	Zhang et al. (2022a)

5-HT, 5-hydroxytryptamine; ATP, adenosine triphosphate; AMPK, AMP-activated protein kinase; BASO, basophil granulocyte; BMDCs, bone marrow-derived dendritic cells; BMDM, bone marrow derived macrophage; CCL2, C-C chemokine ligand 2; CHAT, choline acety transferase; CIS, cisplatin; COX, cyclooxygenase; CXCL2, CXC, motif chemokine ligand 2; DB, C57BLKS/J (−/−) mice; DCs, dendritic cells; DNBS, 2,4,6-dinitrobenzene sulphonic acid; DSS, dextran sulfate sodium; EGCs, enteric glial cells; EMT, epithelial-mesenchymal transition; FGL1, fibrinogen-like protein 1; GLP-1, glucagon-like peptide 1; GM, gut microbiota; HIF-1α, hypoxia-induced factor 1 alpha; HMGB1, high mobility group box 1; IECs, intestinal epithelial cells; IFN, interferon; IL, interleukin; iNOS, inducible nitric oxide synthase; IOs, intestinal organoids; JAK2, Janus kinase 2; JNK, c-Jun N-terminal kinase; lncRNA, long non-coding RNA; LPS, lipopolysaccharides; LYMPH, leukomonocyte; MAPK, mitogen-activated protein kinases; MDA, malondialdehyde; METTL14, Methyltransferase Like 14; MIAT, myocardial infarction associated transcript; miR, microRNA; MLC2, myosin light chain 2; MMP, matrix metalloproteinases; mφs, macrophages; MPO, myeloperoxidase; mRNA, messenger RNA; MUC2, mucin 2; MyD88, myeloid differentiation factor 88; NF-κB, nuclear factor-kappa B; NGF, nerve growth factor; NLRP, NOD-like receptor family pyrin domain containing; Nrf2, nuclear factor erythroid-2-related factor 2; PBMCs, peripheral blood mononuclear cells; PHD2, prolyl hydroxylase domain 2; PI3K, phosphoinostide 3-kinase; PI-IBS, post-infectious irritable bowel syndrome; PPAR-γ, peroxisome proliferator activated receptor gamma; ROCK1, Rho-associated kinase 1; ROS, reactive oxygen species; S1P2, sphingosine-1-phosphate receptor 2; SOD, superoxide dismutase; STAT, signal transducers and activators of transcription; Tfh, T follicular helper cells; Th, T helper; THP-1, human myeloid leukemia mononuclear cells; TJs, tight junction proteins; TLR, toll-like receptor; TNBS, 2, 4, 6-trinitrobenzene sulfonic acid; TNF, tumor necrosis factor; Treg, regulatory T; TSC1, Tuberous sclerosis complex 1; VIP, vasoactive intestinal polypeptide; WLFS, Weight-loaded forced swimming; YAP, Yes-associated protein; ZO-1, Zonula occludens-1.

### 5.2 Modulation of intestinal neurosensory transmission

#### 5.2.1 FGIDs

Patchouli alcohol, one of the active metabolites of *Agastache rugosa* (Fisch. et Mey.) O. Ktze. downregulates excitatory longitudinal muscle myenteric plexus neurons in the distal colon at doses of 5, 10, and 20 mg/kg, decreasing the proportion of Ach- and substance P-positive neurons and the number of Ach- and substance P-positive neurons, downregulating choline acetyltransferase expression, leading to improved symptoms (Chen W. et al., 2022). Previous studies have revealed that capsaicin can activate TRPV1 receptors, and calcitonin gene-related peptide is released from capsaicin; therefore, calcitonin gene-related peptide is thought to be associated with pain, inflammation, and vasodilation (Russell et al., 2014). Liangfu pills are composed of *Alpinia officinarum* Hance and *Cyperus rotundus* L., and network pharmacology and animal experiments have jointly demonstrated that Liangfu pills (1.8, 3.6, and 7.2 g/kg) reduces the levels of TRPV1 and calcitonin gene-related peptide expressions, thereby increasing the gastric emptying rate and small intestinal propulsion in a rat model of gastric cold syndrome for FD treatment. Mosa was used as the positive control (He et al., 2022). TXYF (20 mL/kg) inhibits the activation of enteric glial cells, downregulate the nerve growth factor/tyrosine kinase signaling pathway in the colon of neonatal maternal separation and restraint stress-induced IBS-D rats, reduce the abdominal withdrawal reflex score, and alleviate abdominal pain and diarrhea (Lu and Zhang, 2023). Moreover, electroacupuncture stimulation of “Zusanli” (ST36) reduced the plasma norepinephrine concentration in FD rats, improving impaired gastric slow wave and mediating via the afferent central pathway involving the nucleus of the solitary tract and the vagal cholinergic efferent pathway (Zhang et al., 2020).

#### 5.2.2 IBD

Studies on TCM for modulating sensory transduction in patients with IBD are limited, and only studies on goji berries are being conducted. *Lycium barbarum* L. belongs to the Solanaceae family, and its source of bioactivity is *L. barbarum* polysaccharide isolated from the fruit of *L. barbarum. L. barbarum* L. has the effect of tonifying the liver and kidney, benefiting the essence and brightening the eyes (Chinese Pharmacopoeia Commission, 2020; Tian et al., 2019). *L. barbarum* polysaccharide alone (100 mg/kg) or in combination (50 mg/kg) with capsaicin (6 mg/kg) led to appropriate body weight of DSS-induced UC rats, a reduction in serum IL-6 and colonic TNF-α levels, and a decrease in TRPV1 and transient receptor potential ankyrin 1 expression in colonic tissues for 4 weeks, thereby exerting anti-inflammatory effects; a positive control was not set (Chen Y. S. et al., 2022) (Table 3).

**TABLE 3:** Mechanism of intestinal neurosensory transmission in TCM intervention of FGIDs and IBD.

Type	Disease	Intervention	Main Ingredients/Acupoints	Experiment models	Mechanisms	Reference
FGIDs	FD	Liangfu Pills	Poriferast-5-en-3beta-ol, 1,7-iphenyl-5-hydroxy-3-heptanone, etc.	FD rats	Down-regulating the expression levels of TRPV1/CGRP	He et al. (2022)
					Increasing the expression of 5-HT	
		EA	ST36	FD rats	Improving impaired GSW, mediating via the afferent central pathway involving the NTS and the vagal cholinergic efferent pathway	Zhang et al. (2020)
	IBS-D	Tongxie-Yaofang	Sitosterol, 12-senecioyl-2E,8E,10E-atractylentriol, beta-sitosterol, benzoyl paeoniflorin, etc.	NMS and RS-induced rats	Improving synaptic plasticity through inhibiting the activity of EGCs and the NGF/TrkA signaling pathway in the colon	Lu and Zhang (2023)
		Patchouli alcohol (PA)	Patchouli alcohol (PA)	Chronic restraint stress-induced IBS-D rats	Modulating LMMP excitatory neuron activities, improving intestinal motility and alleviating IBS-induced diarrheal symptoms; decreasing visceral sensitivity; reducing the proportion of excitatory LMMP neurons in the distal colon; decreasing the number of ACh- and SP-positive neurons in the distal colon	Chen et al. (2022a)
					Restoring the levels of ACh and SP in the IBS-D rats	
IBD	UC	Lycium barbarum polysaccharides (LBP) and capsaicin (CAP)	Lycium barbarum polysaccharides (LBP) and capsaicin (CAP)	DSS-induced UC rats	Inhibiting protein expression of TRPV1 and TRPA1	Chen et al. (2022b)
					Inhibiting oxidative stress, proinflammatory cytokines	

ACh, acetylcholine; CGRP, calcitonin gene-related peptide; GSW, gastric slow wave; LMMP, longitudinal muscle myenteric plexus; NMS, neonatal maternal separation; NTS, nucleus of the solitary tract; RS, restraint stress; SP, substance P; TRPV1, transient receptor potential vanilloid 1; TRPA1, transient receptor potential ankyrin 1.

### 5.3 Modulation of the NEI

#### 5.3.1 FGIDs

*Pueraria lobata* (Willd.) Ohwi, a Chinese botanical drug, is often used to treat diarrhea (Chinese Pharmacopoeia Commission, 2020). In a 2-week study using neonatal maternal separation and adult colonic acetic acid stimulation-induced IBS-D rats model, puerarin, a natural metabolite of *Pueraria Mirifica*, inhibited the HPA axis by downregulating CRF, promoting IEC proliferation and repairing the intestinal barrier at doses of 6, 12, and 24 mg/kg, with multipathway therapeutic effects (Wang et al., 2021). Liu et al. (2024) conducted 1-week, 20-min -a-day electroacupuncture in the “Zhongwan” (RN12) and “Zusanli” (ST36) in FD rats and observed that electroacupuncture increased body weight and intestinal propulsion rate, decreased the levels of CRF and CRF-R1 in the hypothalamus and duodenum, and decreased levels of serum corticotropin-releasing hormone and adrenocorticotropic hormone levels in the rat, which demonstrated that electroacupuncture of FD mechanism is related to the CRF signaling pathway. Yu et al. (2019) treated chronic acute combing stress-induced IBS rats with resveratrol (10, 20, and 40 mg/kg) for 22 days at different administration doses, and they discovered that a high dose of resveratrol (40 mg/kg) rescued the decreases in hippocampal PKA, pCREB, and BDNF expression downstream of 5-HT_1_A. However, low resveratrol doses (10 mg/kg) do not have this effect. The modified Liu-Jun-Zi decoction consists of nine botanical drugs, namely, *Codonopsis pilosula* (Franch.) Nannf., *Corydalis yanhusuo* W.T. Wang, *Atractylodes macro cephala* Koidz., *Magnolia officinalis* Rehder and E.H. Wilson, *Aucklandia lappa* DC., *Amomum villosum* Lour., and *G. uralensis* Fisch., which have been shown to downregulate the overexpression of duodenal EC cells and inhibit the signaling of 5-HT_3_ receptor in FD rats (Zhao et al., 2020). Shugan decoction is a proportional preparation of five Chinese botanical drugs, including *Atractylodes macrocephala* Koidz., *Paeonia lactiflora* Pall., *Citrus reticulata* Blanco, *Bupleurum chinense* DC., and *Saposhnikovia divaricata* (Turcz.) Schischk. Serotonin transporter knockout also reduces the frequency and extent of longitudinal smooth muscle contractions in the colon and alleviates diarrhea symptoms in IBS rats (Wang et al., 2020).

#### 5.3.2 IBD

TXYF not only regulates abnormal immunity in patients with UC and improves sensory transduction in patients with IBS but also affects neuroimmunity. By lowering serum 5-HT, TXYF (2.5, 5, and 10 g/kg) can downregulate hepatic 5-HT_2A_ receptor expression, improve hepatic lipid metabolism, and reduce intestinal pathological injury and hepatic steatosis. SASP (0.3 g/kg) was used as a positive control (Zhang X. F. et al., 2022). It can also upregulate the expression of the colonic serotonin transporter to reduce 5-HT levels, reverse decreases in superoxide dismutase activity, and reduce the levels of inflammatory factors such as IL-6 and IL-9 (Luo et al., 2021). Therefore, TXYF, as a commonly used antidiarrheal herbal medicine in clinical practice, can be used to treat FGIDs and IBD through multiple pathways. Shaoyaotang consists of nine Chinese botanical drugs, including *Paeonia lactiflora* Pall., *Areca catechu* L., and Scutellaria *baicalensis* Georgi, which is commonly used in the treatment of dampness–heat diarrhea to clear heat, resolve dampness, and regulate Qi and blood. Shaoyaotang (32 g/kg) upregulates the human serotonin transporter-encoding gene SLC6A4, the key enzyme for 5-HT degradation, monoamine oxidase A and monoamine oxidase B, in DSS-induced UC mice; thus, Shaoyaotang reduces colonic inflammatory cell infiltration by promoting 5-HT transport and degradation (Li Y. N. et al., 2023). In addition, electroacupuncture stimulation of “Zusanli” (ST36) activated Ach release from the VN in the colon to significantly reduce plasma TNF-α, IL-1β, and IL-6 levels, decrease the disease activity index, and increase daily food intake in a rat model of colitis (Jin H. et al., 2019). Herb-partitioned moxibustion of “Qihai” (CV6) and “Tianshu” (ST25) can reduce colonic mucosal congestion and edema in CD rats by downregulating the expression levels of dopamine and dopamine receptor 1 in the colon, hypothalamus, and spinal dorsal horn (Lu et al., 2019) (Table 4).

**TABLE 4:** Mechanism of NEI in TCM intervention of FGIDs and IBD.

Type	Disease	Intervention	Main Ingredients	Experiment models	Mechanisms	Reference
FGIDs	FD	Xiaoerfupi (XEFP)	Taraxerone, N-Methyltyramine, Ergotamine, Cis-Geraniol, etc.	Administration of iodoacetamide (IA) and interval fasting-induced FD rats	Regulating the HTR_3_A and c-FOS	Ji et al. (2019)
		Modified Liu-Jun-Zi (MLJZ)	D-Isoboldine, β-Ionone, α-Curcumene, Licoflavone A, Obovatol, Cotunolide	Iodoacetamide gavage and tail clamping method-induced FD rats	Alleviating visceral hypersensitivity in FD by regulating EC cell-5HT_3_r signaling in duodenum	Zhao et al. (2020)
		Paeoniflorin	Paeoniflorin	Iodoacetamide or clonidine-induced FD rats	Inhibiting the AChE activity; increasing the levels of ACh and ghrelin	Zou et al. (2020b)
					Up-regulating the expression of adhesion proteins (occludin and ZO-1)	
		EA	RN12, ST36	Sequential tail clamping, an irregular diet, and ice water gavage-induced FD rats	Decreasing the levels of CRF and CRF-R1 in the hypothalamus and duodenum, and decreasing serum corticotropin-releasing hormone and adrenocorticotropic hormone levels	Liu et al. (2024)
	IBS-D	Cinnamon extract	Procyanidin B1/B2, catechin, cinnamic acid, cinnamyl alcohol, cinnamic aldehyde	MS IBS-D rats; TNBS-induced post-inflammatory IBS-D rats	Inhibiting Tph1 expression and controlling 5-HT synthesis	Yu et al. (2022a)
		Puerarin	Puerarin	NMS and ACAAS-induced rats	Inhibiting activity of the HPA axis by the suppressed expression of CRF	Wang et al. (2021)
					Enhancing the proliferation of colonic epithelial cells by up regulating the expression of p-ERK/ERK and repairing the colonic mucus barrier by up regulating occludin expression	
	IBS	Shugan decoction (SGD)	SaikosaponinA, paeoniflorin, 5-O-Methylvisammioside, hesperidin, and cimicifugoside	SERT-KO rats	Down regulating M_3_R expression in the colon	Wang et al. (2020)
		Resveratrol	Resveratrol	CACS-induced IBS rats	Regulating 5-HT_1_A-dependent PKA-CREB-BDNF signaling in the brain-gut axis	Yu et al. (2019)
					Improving anti-IBS-like effects on depression, anxiety, visceral hypersensitivity and intestinal motility abnormality	
IBD	UC	*Centella asiatica*	Asiaticoside	DSS-induced mice	Promoting c-Kit expression in the colon and 5-HT in the brain	Li et al. (2021a)
					Up-regulating the expression of tight junction protein (ZO-1, E-cadherin), suppressing inflammatory cell infiltration with decreased MPO activity in the colon	
		Shaoyaotang (SYT)	Quercetin, kaempferol β- Sitosterol, etc.	DSS-induced mice	Regulating 5-HT degredation pathways	Li et al. (2023b)
		Tongxie Yaofang	Sitosterol, 12-senecioyl-2E,8E,10E-atractylentriol, beta-sitosterol, benzoyl paeoniflorin, etc.	TNBS/ethanol solution enema + restraint stress + diet loss-induced liver depression and spleen deficiency UC rats	Up regulating the expression of SERT in the colon to reduce the content of 5-HT; reducing the content of 5-HT and the expression of 5-HT_2A_R in the liver	Luo et al. (2021)
					Increasing the level of SOD	
					Improving hepatic 5-HT_2A_R expression via regulation of the peripheral 5-HT	Zhang et al. (2022d)
					Changing the level of hepatocyte autophagy via stabilization of the hepatic lipid metabolism	
		EA	ST36	TNBS-induced UC rats	Inhibiting the release of pro-inflammatory cytokines by improving sympathetic-vagal imbalance	Jin et al. (2019b)
	CD	HPM	CV6, ST25	TNBS-induced CD rats	Down regulating DA and D_1_R concentrations as well as D_1_R mRNA expression	Lu et al. (2019)

5HT_2A_R, 5-hydroxytryptamine 2A receptor; 5HT_3_r, 5-hydroxytryptamine 3 receptor; ACAAS, adult colonic acetic acid stimulation; AChE, acetylcholinesterase; BDNF, brain derived neurotrophic factor; CACS, chronic acute combing stress; c-FOS, proto-oncogene c-Fos; CREB, cAMP-response element binding protein; CRF, corticotropin-releasing receptor; D_1_R, dopamine receptor 1; DA, dopamine; EC, enterochromaffin cell; HPA, hypothalamic-pituitary-adrenal; HPM, herb-partitioned moxibustion; HTR_3_A, 5-hydroxytryptamine receptor 3A; M_3_R, muscarinic receptor subtype-3; MS, maternally separated; SERT, serotonin transporter; SERT-KO, serotonin transporter-knockout; Tph1, Tryptophan Hydroxylase 1.

### 5.4 Regulation of intestinal microbial homeostasis

#### 5.4.1 FGIDs

Members of the Magnoliaceae family M. *officinalis* Rehd. et Wils. are used for the treatment of bloating and indigestion because of their ability to regulate gastrointestinal Qi (Chinese Pharmacopoeia Commission, 2020). As one of the extracts of M. *officinalis* Rehd. et Wils, magnoloside A (5, 10, and 20 mg/kg) increased the abundance of intestinal microbiota; however, magnoloside A decreases the abundance of beneficial intestinal bacteria, such as *Akkermansia*, at high concentrations of magnolinoside A (20 mg/kg) (Xue et al., 2019). Atractyloside-A, one of the main metabolites of the Chinese medicine *Atractylodes lancea* (Thunb.) DC., can exert therapeutic effects by decreasing the Firmicutes/Bacteroidetes ratio and downregulating the expression of 5-HT and 5-HT_3_ receptors (Xu J. et al., 2022). One-week treatment with acupuncture or moxibustion of bilateral “Zusanli” (ST36) in IBS-D rats decreased the relative abundance of Bacteroidetes and Proteobacteria, increased the relative abundance of Firmicutes, decreased the synthesis of lipopolysaccharides, and attenuated inflammatory response. In addition, the moxibustion group promotes the synthesis and metabolism of amino acids, such as tyrosine and tryptophan (Lai et al., 2023). Fuzi-Lizhong pills (50 and 150 mg crude drug/mL) have been used to treat digestive disorders and increase the abundance of *Lactobacillus*, a key flora for repairing the immune barrier of the intestinal tract, decrease the abundance of inflammation-associated microbiota such as *Bacteroide,s* and significantly decrease the levels of TNF, IL-1β, IL-6, and INF-γ and attenuate diarrhea in IBS-D rats in the state of spleen yang deficiency (Zhen et al., 2021).

#### 5.4.2 IBD

The regulatory effect of Chinese medicine on IBD flora focuses on the restoration of the normal composition of intestinal flora by reducing the abundance of harmful bacteria, increasing the levels of beneficial bacteria, and regulating microbiota metabolism. Huai Hua San is based on *Sophora japonica* L., which is widely used for treating lower gastrointestinal diseases, and an 8-day study showed that Huai Hua San can reduce the Firmicutes/Bacteroidetes ratio to that of healthy people, restore colonic vascular permeability, and reduce the disease activity index (Liu P. et al., 2020). Pulsatilla decoction, which consists of four Chinese botanical drugs, namely, *Pulsatilla chinensis* (Bunge) Regel, *Coptis chinensis* Franch., *Phellodendron chinense* Schneid., and *Fraxinus rhynchophylla* Hance, originated in the Eastern Han Dynasty. It can increase body weight and colon length in DSS-induced UC mice by potentially repairing the intestinal mucosal barrier through the upregulation of tight junction proteins such as zonula occludens-1 and occludin. This alteration leads increases the relative abundance of Bacteroidetes, reductions in the relative abundance of Firmicutes and Proteobacteria, and elevations in the total content of SCFAs in the intestines, with an optimal dose of 8.1 g/kg (Niu et al., 2023). In addition, electroacupuncture of “Dachangshu” (BL25) and bilateral “Tianshu” (ST25) in obese IBD rats also reduce the Firmicutes/Bacteroidetes ratio (Yang et al., 2024). The methanol extract of *Schizonepetae Spica* (500 and 1,000 mg/kg) improved the intestinal flora by downregulating the abundance of harmful bacteria such as *Clostridiales* and *Desulfovibrio* and upregulated the abundance of beneficial bacteria such as *Muribaculaceae* and *Ligolactobacillus* in DSS-induced colitis mice. Salazosulfapyridine (1,000 mg/kg) was used as the positive control (Ye et al., 2023).

*Schisandra chinensis* polysaccharides significantly downregulated the levels of IL-6, IL-10, IL-17, and TNF-α; antagonized DSS-induced intestinal dysbiosis in mice; increased the levels of acetic acid, propionic acid, and total SCFAs; and improved SCFAs metabolism, which is useful for treating the symptoms of abdominal pain and blood in the stool of mice. Salazosulfapyridine (200 mg/kg) was used as the positive control (Su et al., 2020). Paeonol similarly restores the homeostasis of the intestinal flora and regulates SCFAs metabolism (Zhao M. et al., 2023). Recent metabolomics and animal studies have shown that uric acid levels are closely related to the integrity of the intestinal barrier and that abnormally high uric acid levels in the intestinal tract can lead to deterioration of the intestinal barrier, whereas one of the extracts of *Rheum palmatum* L., rhein (50 and 100 mg/kg) indirectly affected purine metabolism by increasing the abundance of intestinal lactobacilli in mice with DSS-induced chronic colitis, lowering the concentration of intestinal uric acid, and reversing the increase in the permeability of the intestinal barrier in IBD. A positive control was not set (Wu et al., 2020) (Table 5).

**TABLE 5:** Mechanism of microbial homeostasis in TCM intervention of FGIDs and IBD.

Type	Disease	Intervention	Main Ingredients/Acupoints	Experiment models	Mechanisms	Reference
FGIDs	FD	Magnoloside A (MA)	Magnoloside A (MA)	Transient neonatal gastric irritation and mature ADF-induced FD rats	Modulating the secretion of related brain-gut peptides	Xue et al. (2019)
					Altering the composition of intestinal microbiota	
	IBS-D	Fuzi-Lizhong pill (FLZP)	Liquiritin, glycyrrhizin, benzoylmesaconine, benzoylaconine, benzoylhypaconine	IBS-D rats in the state of Spleen–Yang deficiency	Affecting bacterial diversity and community structures in the host	Zhen et al. (2021)
		Atractyloside-A	Atractyloside-A	Folium sennae-induced SDD mice	Regulating the TLR4/MyD88/NF-κB signaling pathway, which inhibited inflammation and regulated the intestinal flora	Xu et al. (2022a)
		Acupuncture or moxibustion	ST36	Acetic acid enema combined with binding tail-clip stress-induced IBS-D rats	Decreasing the relative abundance of *Bacteroidetes* and *Proteobacteria*, increasing the relative abundance of *Firmicutes*, decreasing the synthesis of lipopolysaccharides	Lai et al. (2023)
	IBS	Shugan Decoction (SGD)	SaikosaponinA, Paeoniflorin, 5-O-Methylvisammioside, Hesperidin, Cimicifugoside	WAS-induced IBS-D model rats	Regulating specific intestinal microbiota and some metabolic pathways	Hang et al. (2022)
		Chang-Kang-Fang Formula	Paeoniflorgenone, beta-sitosterol, sitosterol, matrine, etc.	CACS-induced rats	Inducing structural changes in the gut microbiota; decreasing the F-B ratio, and the abundances of *Corynebacteriales* and *Clostridiales*; increasing the levels of *Lactobacillus*; providing beneficial effects against intestinal tract motility, high visceral sensitivity, and behavioral abnormalities through the microbiota-gut-brain axis	Ling et al. (2022)
IBD	UC	Rhein	Rhein	DSS-induced chronic colitis mice	Modulating gut microbiota, indirectly changing purine metabolism; altering gut microbiota composition and increasing *Lactobacillus* level leading to decrease uric acid levels	Wu et al. (2020)
		Lycium barbarum Glycopeptide (LbGP)	Lycium barbarum Glycopeptide (LbGP)	DSS-induced UC mice	Modulating the composition of microbial communities	Huang et al. (2022b)
		EtOAc fractions of H. attenuatum Choisy (Ha-EtOAc)	EtOAc fractions of H. attenuatum Choisy (Ha-EtOAc)	DSS-induced UC mice; LPS-induced RAW264.7 macrophage		Jin et al. (2019a)
		Huai hua san (HHS)	Quercetin, kaempferol, naringin and rutin	DSS-induced UC rats	Reducing colitis-associated high increased ratio of Bacteroidetes to Firmicutes to a normal level	Liu et al. (2020b)
		Berberine (BBR)	Berberine (BBR)	DSS-induced colitis mice	Regulating intestinal immune cell differentiation by affecting the growth of *B. fragilis*	Zheng et al. (2021)
		Si-Ni-San (SNS)	Gallic acid, Catechin, Chlorogenic acid, Liquiritin, albiflorin, etc.	DSS-induced acute colitis mice	Decreasing abundance proinflammatory species, upregulating abundance of anti-inflammatory species	Cai et al. (2023)
					Altering microbiota metabolite metabolism; on favoring the growth of potential probiotics	
		Patrinia villosa Juss. (P.V)	Phenylpropanoids, Flavonoids, Terpenes, Saponins, etc.	TNBS-induced UC rats	Exerting anti-inflammatory effect by impacting bile acid levels, activating VDR	Wang et al. (2022a)
					Inhibiting the overactivation of NF-κB signaling pathways	
		Ginsenoside Rg1	Ginsenoside Rg1	DSS-induced acute colitis mice	Regulating gut microbiota composition	Cheng et al. (2022b)
					Regulating microbial tryptophan metabolism	
		Gegen Qinlian decoction (GQD)	Berberine, baicalin, and puerarin, etc.	DSS-induced colitis mice	Regulating gut microbiota-related tryptophan metabolism	Wang et al. (2023a)
					Restoring the generation of indole derivatives to activate AhR-mediated IL-22 production	
		Atractylodes macrocephala Koidz. (AM)	Atractylodes macrocephala Koidz. volatile oil (AVO)	DSS-induced acute UC mice	Decreasing potentially harmful bacteria	Cheng et al. (2023)
					Enriching potentially beneficial bacteria; altering gut microbiota metabolism by regulating 56 gut microbiota metabolites involved in 102 KEGG pathways; maintaining intestine homeostasis by many metabolism pathways: amino acid metabolism (especially tryptophan metabolism), bile acids metabolism, and retinol metabolism	
		Processed Vladimiriae Radix (pVR) and raw Vladimiriae Radix (rVR)	Costunolide (COS), dehydrocostus lactone (DEH)	DSS-induced UC rats	Modulating the structure of gut microbiota; reversing the reduced abundance of intestinal flora; correcting increase of cytidine, N6-acetyl-L-lysine and β-alanine, and decrease of 2-Aminonicotinic acid	Yu et al. (2021)
					Regulating the disorder of metabolites and their related metabolic pathways which contributed to the development of colitis	
		Lizhong decoction (LZD)	Gingerols, Ginsenosides, Atractylone, Orange linoleum, Elemene, Isoeugenol, Glycyrrhetic acid, β-sitosterol, flavonoids, and Hydroxycoumarins	DSS-induced colitis mice	Increasing species number, restoring the richness	Zou et al. (2020a)
					Decreasing the expression of IL-6, TNF-α (except L-rVR) and IL-10	
		Paeonol (pae)	2′-hydroxy- 4′-methoxy acetophenone	DSS-induced UC mice	Increasing *C. butyricum* and SCFAs production	Zhao et al. (2023b)
		The methanol extract of Schizonepetae Spica (JJSM)	Luteolin, eriodictyol, fisetin, and kaempferol	DSS-induced colitis mice	Improving intestinal flora with down-regulating the abundance of harmful bacteria such as *Clostridiales* and *Desulfovibrio* and up-regulating the abundance of beneficial bacteria such as *Muribaculaceae* and *Ligolactobacillus*	Ye et al. (2023)
					Enhancing the production of SCFAs	
		Schisandra chinensis polysaccharide (SCP)	D-glucosamine, rhamnose, glucose, D-galactose, D-xylose and D-arabinose	DSS-induced UC mice	Regulating the imbalance of gut microbiota	Su et al. (2020)
					Increasing the content of SCFAs; regulating metabolism of gut microbiota	
		Pulsatilla decoction (PD)	Aesculin, Aesculetin, Jateorhizine Hydrochloride, Palmatine chloride, Berberine, Pulsatilasaponin B4	DSS-induced UC mice	Maintaining the homeostasis and diversity of gut microbiota	Niu et al. (2023)
					Increasing the content of SCFAs	
					Repairing the colonic mucosal barrier	
		The main active components of Jiawei Gegen Qinlian Decoction (PBM)	Puerarin, Baicalein, Berberine, Glycyrrhiic acid, Magnolol	DSS-induced UC rats	Regulating the gut microbiota by increasing *Akkermansia* and *Romboutsia*, decreasing *Escherichia-Shigella*	Li et al. (2021b)
					Increasing the production of propionate and total SCFAs, regulating medium and long chain fatty acids (M-LCFAs), maintain bile acids (BAs) homeostasis, and regulating amino acids (AAs) metabolism	
	IBD	EA	BL25, ST25	HFD-induced IBD mice	Decreasing the Firmicutes/Bacteroidetes ratio	
					Activating the Nrf2 signaling pathway; inhibiting intestinal inflammation and ferroptosis	

ADF, alternate-day fasting; AhR, aryl hydrocarbon receptor; F-B, Firmicutes-Bacteroidetes; HFD, high-fat diet; KEGG, kyoto encyclopedia of genes and genomes; SCFAs, short-chain fatty acids; SDD, spleen deficiency diarrhea; VDR, Vitamin D receptor; WAS, water avoid stress.

## 6 Conclusions

**FIGURE 3:**
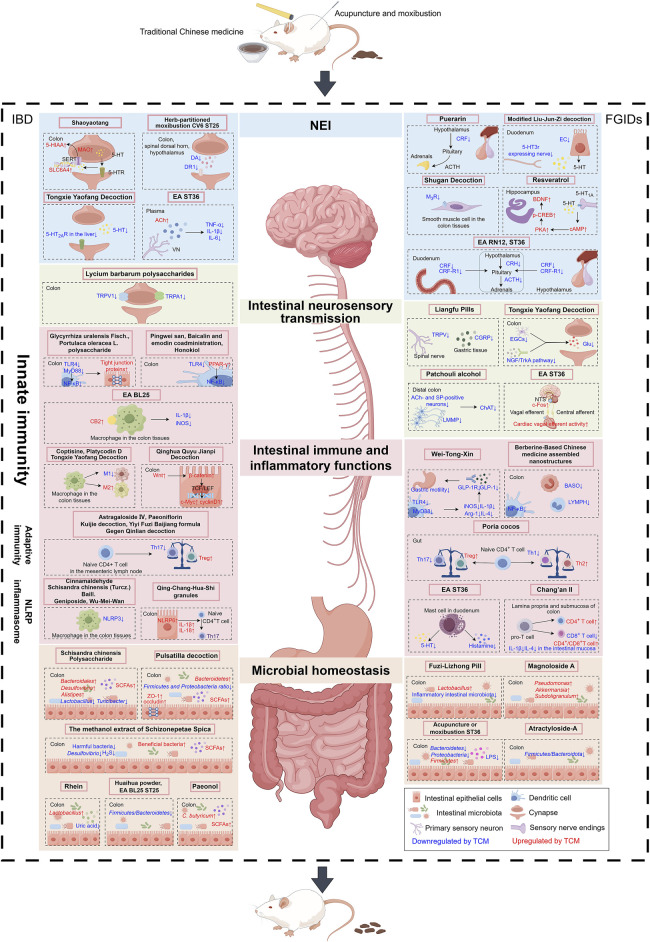
Mechanism of TCM intervention of FGIDs and IBD based on the brain-gut axis theory (by Figdraw). TCM, traditional Chinese medicine; IBD, inflammatory bowel disease; FGIDs, functional gastrointestinal disorders; NEI, neuroendocrine-immune network; 5-HT, 5-hydroxytryptamine; 5-HIAA, 5 hydroxyindoleacetic acid; MAO, monoamine oxidase; SERT, serotonin transporter; 5-HT_2A_R, 5-hydroxytryptamine 2A receptor; DA, dopamine; DR1, dopamine receptor 1; EA, electroacupuncture; ACh, acetylcholine; VN, vagus nerve; TNF, tumor necrosis factor; IL, interleukin; TRPV1, transient receptor potential vanilloid 1; TRPA1, transient receptor potential ankyrin 1; TLR4, toll-like receptor 4; NF-κB, nuclear factor -kappa B; MyD88, myeloid differentiation factor 88; PPAR-γ, peroxisome proliferator activated receptor gamma; iNOS, inducible nitric oxide synthase; Th, T helper; Treg, regulatory T; NLRP, NOD-like receptor family pyrin domain containing; SCFAs, short-chain fatty acids; ZO-1, Zonula occludens-1; CRF, corticotropin-releasing factor; ACTH, adrenocorticotropic factor; EC, enterochromaffin cell; 5-HT_3_R, 5-hydroxytryptamine 3 receptor; M_3_R, muscarinic receptorsubtype-3; BDNF, brain derived neurotrophic factor; p-CREB, p-cAMP-response element binding protein; CRH, corticotropin-releasing hormone; CGRP, calcitonin gene-related peptide; ChAT, choline acetyltransferase; EGCs, enteric glial cells; NGF, nerve growth factor; Glu, glutamate; SP, substance P; LMMP, longitudinal muscle myenteric plexus; c-Fos, proto-oncogene c-Fos; NTS, nucleus of the solitary tract; GLP-1, glucagon-like peptide 1; Arg-1, arginase-1; BASO, basophil granulocyte; LYMPH, leukomonocyte; LPS, lipopolysaccharides.

The pathogenesis of FGIDs and IBD involve a complex interplay of mechanisms with the brain–gut axis, encompassing inflammatory responses, immune dysregulation, impaired neurosensory transmission, disrupted neuroendocrine–immune interactions, and alterations in the composition of the gut microbiota. TCM shows effectiveness in alleviating disease symptoms, slowing disease advancement, and averting disease reappearance. According to the above TCM that can treat FGIDs and IBD (Figure 3), terpenes (triterpenoid of *Poria cocos*, patchouli alcohol, and platycodon D, etc.), polysaccharides (water-soluble polysaccharide of *P. cocos,* acidic polysaccharide of *P. cocos, Portulaca oleracea L.* polysaccharide, *Lycium barbarum* polysaccharide, etc.), flavonoids (baicalin, Puerarin, etc.), glycosides (paeoniflorin, magnoloside A, Atractyloside-A, etc.), and other metabolites (alkaloids, phenols, anthraquinones, aldehydes, etc.) were major bioactive metabolites in TCM for the treatment of FGIDs and IBD. *Schisandra chinensis* (Turcz.) Baill., Wei-Tong-Xin, Shaoyaotang, TXYF, and QCHS, etc., were the main TCM botanical drugs and formulas. Acupuncture treats FGIDs and IBD primarily by electroacupuncture stimulation of “Zusanli” (ST36) and herb-partitioned moxibustion of “Qihai” (CV6) and “Tianshu” (ST25). The mechanism of TCM interventions on FGIDs and IBD is multipathway, namely, botanical drugs, metabolites, prescriptions, and acupuncture. However, the greatest number of studies has focused on the regulation of intestinal inflammation and immunity and the fewest on intestinal neurosensory transmission, and acupuncture mainly focuses on modulating the NEI.

We have outlined the potential molecular mechanisms of TCM in FGIDs and IBD, with focus on therapeutic outcomes, targets, and signaling pathways in animal models: 1) modulation of the levels of proinflammatory cytokines in the intestinal tract, such as ILs and tumor necrosis factor, downregulation of proinflammatory signaling pathways such as NF-κB, MAPK, and levels of NLRP inflammatory vesicles; 2) antagonism of the aberrant activation of immune cells in the intestinal tract, such as M2-like polarization, downregulation of the Th1/Th2 and Th17/Treg cell ratios, and downregulation of TLR levels on the immune cell surface; 3) restoration of the intestinal barrier and promotion of the expression of tight junction proteins, such as zonula occludens-1 and occludin; 4) intervention in visceral hypersensitivity reaction by decreasing the number of hypersensitivity-associated neurons and osmosensory channel expression, such as TRPV; 5) modulation of the levels of intestinal barrier injury-related neurotransmitters (Ach, 5-HT, and DA) and downregulation of CRF levels to antagonize their pathogenic effects; and 6) upregulation of the abundance of beneficial bacteria in the intestine and the downregulation of that of detrimental bacteria, e.g., decreasing the Firmicutes/Bacteroidetes ratio and increasing the abundance of *Lactobacillus,* to restore the normal intestinal metabolism, e.g., SCFAs metabolism and purine metabolism. Therefore, TCM can elicit therapeutic effects by targeting multiple pathways, components, and modes of action, indicating its promising developmental potential.

This paper discussed FGIDs and IBD, a large group of diseases with unclear pathogenesis and no clear cure is available, which are urgent problems for modern medicine. TCM has shown promise in treating FGIDs and IBD through evidence-based practices guided by a holistic approach. For example, 4 weeks of TXYF treatment in patients with IBD effectively reduced the number of diarrhea episodes, improved fecal character, and was superior to the antispasmodic pinaverium (Fan et al., 2017). Acupuncture and moxibustion were reported to alleviate symptoms in patients with mildly to moderately active CD and repair the intestinal barrier (Bao et al., 2022). TCM targets the brain–gut axis by modulating intestinal immunity and inflammation, normalizing sensory transmission to reduce visceral hypersensitivity, regulating neuroimmunity, and restoring intestinal microbial balance. The review of research on TCM treatment of FGIDs and IBD, the following problems have been identified: 1) The small sample size of clinical studies and unreported inclusion and exclusion criteria make it difficult to avoid selection bias, so the credibility of the experimental results cannot be guaranteed, and most clinical research evidence is of poor quality. 2) Because FGIDs and IBD are chronic recurrent diseases, most clinical studies have a short follow-up period; thus, longer follow-up studies are needed to better observe the long-term efficacy of TCM treatments. 3) Research on existing mechanism focuses on TCM regulation against a single target, lack of multi-target, multi-faceted validation, and the need to evaluate the synergistic therapeutic effect of different components of TCM formulas to regulate multiple targets in the direction of efforts. 4) Insufficient depth of mechanistic studies on TCM and the need for more rigorous design in terms of the active metabolite and effective concentration to confirming the safety and effectiveness of the potentially effective metabolite. 5) The core of TCM is the overall concept and treatment based on pattern differentiation, and existing animal models of the two diseases do not correspond with the TCM evidence patterns, and TCM treatments deviated.

The goal of modern medicine in IBD treatment has gradually shifted to the promotion of intestinal mucosal healing and long-term relief of clinical symptoms (Turner et al., 2021). Existing IBS treatments also fail to meet the clinical need for pain relief, and the recurrence of FGID symptoms is also of concern (Ford et al., 2020b; Singh et al., 2022). The treatment of TCM in these aspects has advantages. A meta-analysis showed that TCM retention enema was superior to conventional drug therapy in terms of clinical efficacy and reduction of recurrence and colonoscopic improvement of ulceration in patients with UC (Yan et al., 2021). Moreover, the treatment of IBS and FD with Weichang’an pill combined with Western medicine is superior to the application of Western medicine alone, and the combined treatment of TCM and Western medicine can significantly alleviate abdominal pain and bloating and reduce FD recurrence without increasing the incidence of adverse reactions (Jiabao et al., 2023). This proves the potential of TCM. However, the mechanism of action of TCM is not clear; thus, it lacks credibility for large-scale application in clinical treatment. Moreover, hepatotoxicity and drug–drug interactions of TCM should be noted. *Polygonum multiflorum* Thunb., *Scutellaria baicalensis* Georgi, and *Gynura* segetum are the three most commonly reported to cause drug-induced liver injuries (Ballotin et al., 2021), Salvia miltiorrhiza Bge. Increases the risk of bleeding when taken with warfarin (Chan, 2001). In recent years, the concept of brain–gut–microbiota axis has received widespread attention and has been applied to various diseases, which is a promising development direction (Zhu et al., 2024). Research on gut microbiota by TCM is still immature. Therefore, more studies are needed to fully substantiate the efficacy of TCM in restoring gut microbial homeostasis. Care prevent and cure diseases, the modernization and standardization of TCM are desired to. With the scientists’ deepening of the concept of brain–gut axis and the continuous development of science and technology, as well as the interpenetration and integration of TCM and modern medicine, a breakthrough may occur in the prevention and treatment of FGIDs and IBD by TCM, which will bring the hope of cure to more patients.

One limitation of our study is that we employed a narrative review approach instead of a systematic review, which inherently comes with certain drawbacks. Specifically, the scope of literature we reviewed was not as comprehensive as it could have been, potentially leading to an incomplete representation of the topic. This lack of exhaustive literature retrieval suggests that readers should interpret the conclusions of this paper with caution. While narrative reviews can offer valuable insights, they do not always provide clear transparency regarding the criteria used for selecting and excluding literature, which may introduce biases or omissions. We acknowledge these limitations and advise readers to consider them when evaluating the conclusions drawn in this study.

## Figures and Tables

**Table:** 

**FGIDs**	functional gastrointestinal disorders
**IBD**	inflammatory bowel disease
**TCM**	traditional Chinese medicine
**IBS**	irritable bowel syndrome
**FD**	functional dyspepsia
**UC**	ulcerative colitis
**CD**	Crohn’s disease
**ENS**	enteric nervous system
**CNS**	central nervous system
**NEI**	neuroendocrine-immune network
**Th**	T helper
**TLR**	toll-like receptor
**VN**	vagus nerve
**TRPV**	transient receptor potential vanilloid
**Piezo**	piezo-type echanosensitive ion channel component
**5-HT**	5-hydroxytryptamine
**EC**	enterochromaffin cell
**IBS-D**	diarrhea predominant-irritable bowel syndrome
**HPA**	hypothalamic-pituitary-adrenal
**CRF**	corticotropin-releasing factor
**SCFAs**	short-chain fatty acids
**IECs**	intestinal epithelial cells
**DCs**	dendritic cells
**pDCs**	plasmacytoid dendritic cells
**Treg**	regulatory T
**NLRP**	NOD-like receptor family pyrin domain containing
**DSS**	dextran sulfate sodium
**Ach**	acetylcholine
**TXYF**	Tong-Xie-Yao-Fang
**QCHS**	Qing-Chang-Hua-Shi
**NF-κB**	nuclear factor-kappa B
